# Supplementary material for: Nanocurvature-induced field effects enable control over the activity of single-atom electrocatalysts
Source: Nat Commun. 2024 Feb 26;15:1719. doi: 10.1038/s41467-024-46175-1 (PMC10897157; doi:10.1038/s41467-024-46175-1)
Supplement: Supplementary file 1 — Supplementary Information [file 41467_2024_46175_MOESM1_ESM.pdf]

Supplementary Information

Nanocurvature-induced field effects enable control over the activity of single-atom electrocatalysts

Bingqing Wang[1,7], Meng Wang[1,2,3,7], Ziting Fan[1], Chao Ma[4], Shibo Xi[5], Lo-Yueh Chang[6], Mingsheng Zhang[2], Ning Ling[1], Ziyu Mi[5], Shenghua Chen[4], Wan Ru Leow[5], Jia Zhang[3], Dingsheng Wang[4] and Yanwei Lum[1,2]*

[1]Department of Chemical and Biomolecular Engineering, National University of Singapore, Singapore, Singapore

[2]Institute of Materials Research and Engineering, Agency for Science, Technology and Research (A*STAR), Innovis, Singapore

[3]Institute of High Performance Computing, Agency for Science, Technology, and Research (A*STAR), Connexis, Singapore

[4]Department of Chemistry, Tsinghua University, China

[5]Institute of Sustainability for Chemicals, Energy and Environment, Agency for Science, Technology and Research (A*STAR), Jurong Island, Singapore

[6]National Synchrotron Radiation Research Centre, Hsinchu, Taiwan

[7]These authors contributed equally to this work

*Corresponding author: lumyw@nus.edu.sg

Contents

Supplementary Figures

Supplementary Tables

Supplementary References

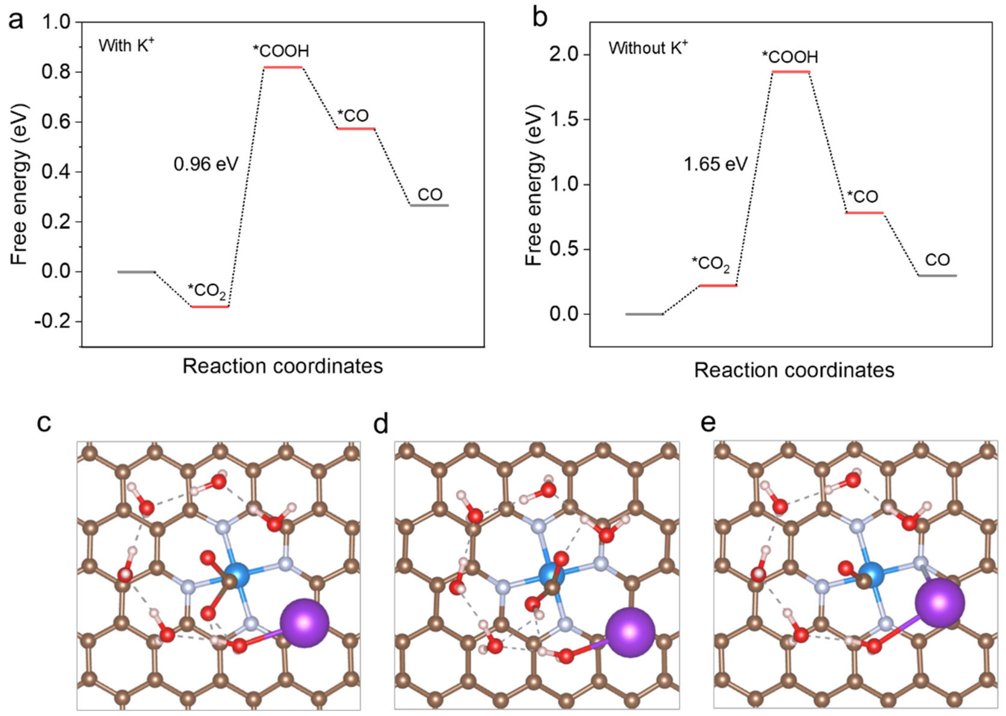

Supplementary Fig. 1 | Reaction pathway Gibbs free energy diagrams of Ni-N_4 in the (a) presence of K^+ and (b) absence of K^+. The free energy change of the rate-limiting step is shown in each case. Structure models of Ni-N_4 with K^+ and H_2O adsorbed with the intermediates: (c) *CO_2, (d) *COOH, and (e) *CO. The brown, gray, blue, red, purple, and light pink spheres represent carbon, nitrogen, nickel, oxygen, potassium, and hydrogen atoms respectively.

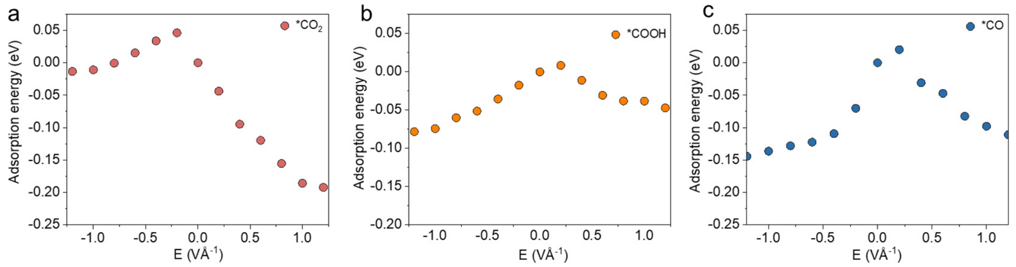

Supplementary Fig. 2 | Adsorption energies of CO_2R intermediates as a function of electric field strength on Ni-N_4: (a) *CO_2, (b) *COOH and (c) *CO.

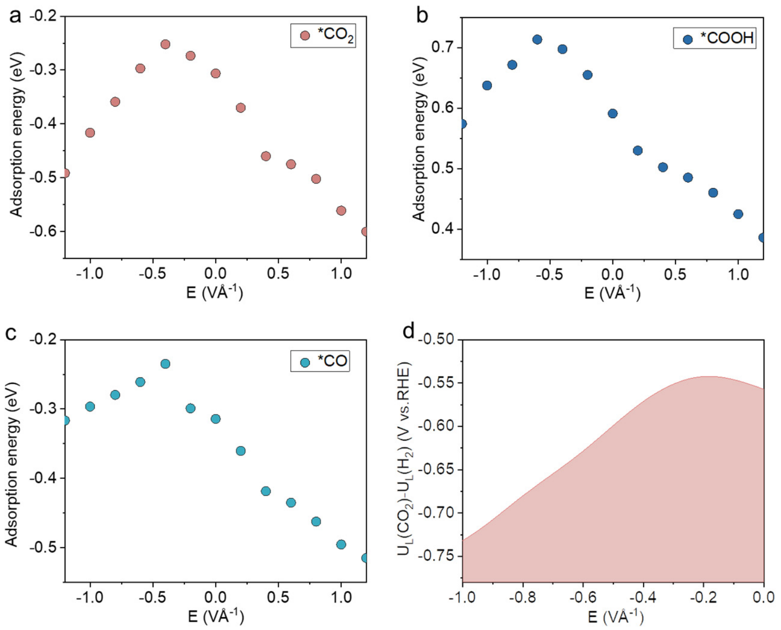

Supplementary Fig. 3 | Adsorption energies of CO_2R intermediates as a function of electric field strength on Fe-N_4: (a) *CO_2, (b) *COOH and (c) *CO. (d) theoretical limiting potential difference of $U_L(CO_2)$-$U_L(H_2)$ on Fe-N_4 as a function of interfacial electric field strength.

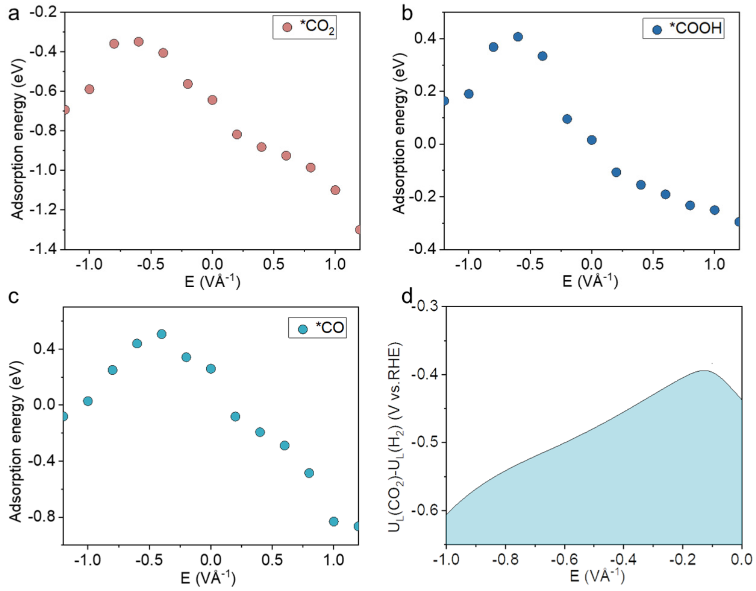

Supplementary Fig. 4 | Adsorption energies of CO_2R intermediates as a function of electric field strength on Co-N_4: (a) *CO_2, (b) *COOH and (c) *CO. (d) theoretical limiting potential difference of $U_L(CO_2)$-$U_L(H_2)$ on Co-N_4 as a function of interfacial electric field strength.

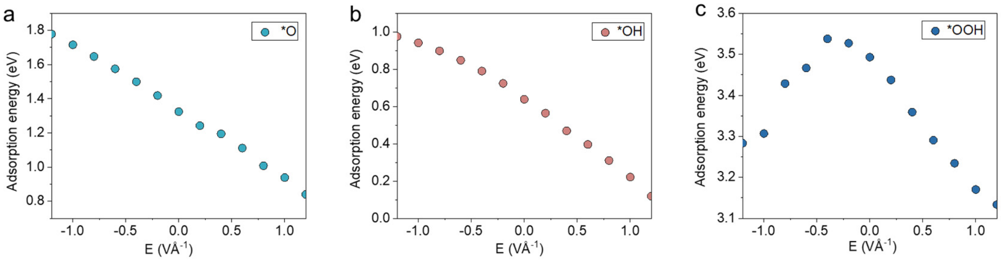

Supplementary Fig. 5 | Adsorption energies of ORR intermediates as a function of electric field strength on Fe-N_4: (a) *O, (b) *OH and (c) *OOH.

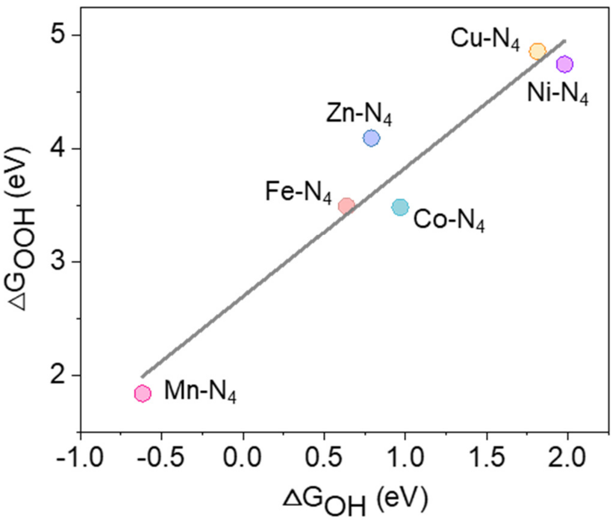

Supplementary Fig. 6 | Calculated scaling relationship between ΔG_{OH} and ΔG_{OOH} for M-N_4 SACs. A slope value of 1.14 was obtained, which is close to the theoretical value of 1.

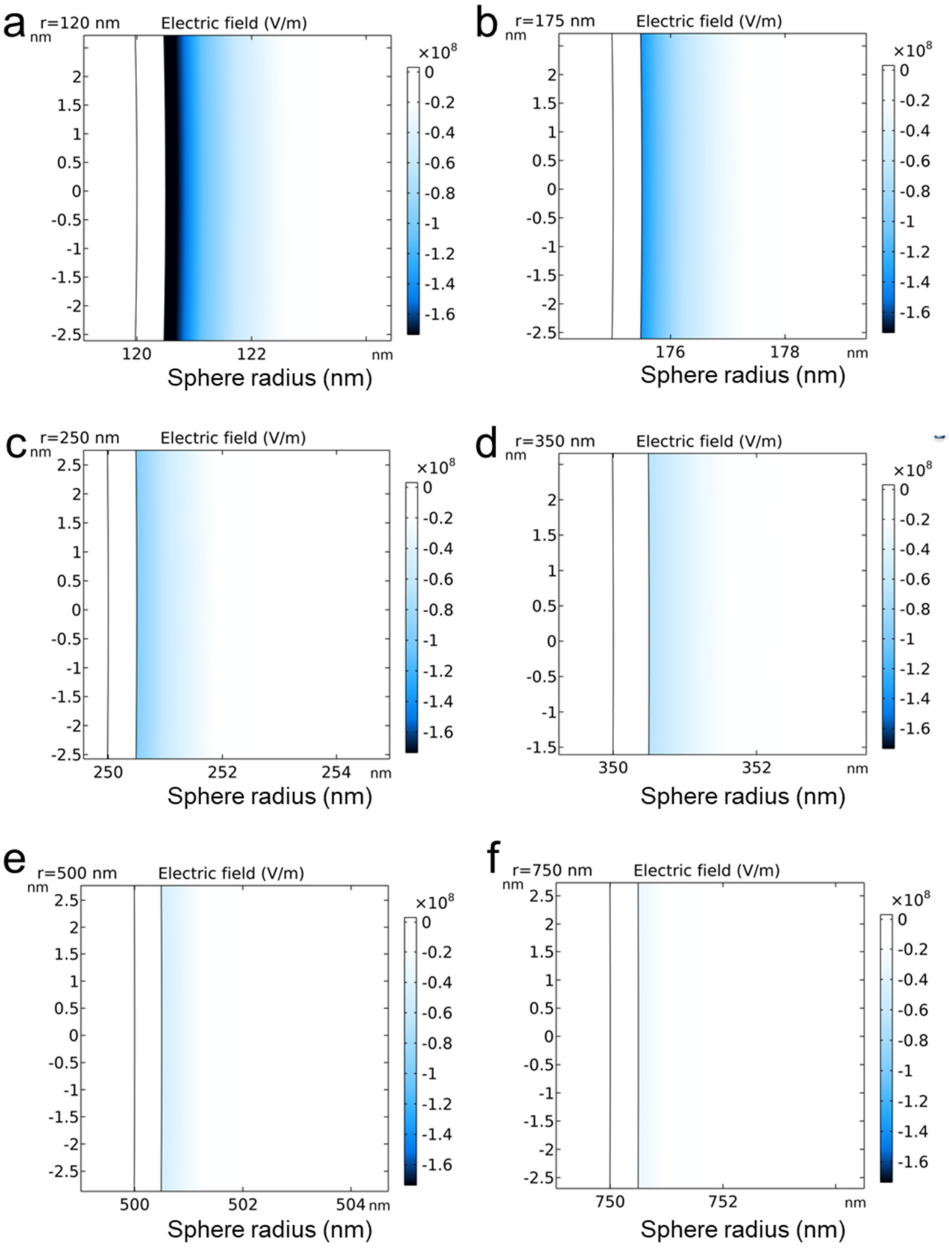

Supplementary Fig. 7 | Computed interfacial electric field strength from finite-element numerical method simulations on various carbon sphere support diameters. (a) 240 nm, (b) 350 nm, (c) 500 nm, (d) 700 nm, (e) 1000 nm and (f) 1500 nm. Note: the sphere radius rather than diameter is shown in the figure.

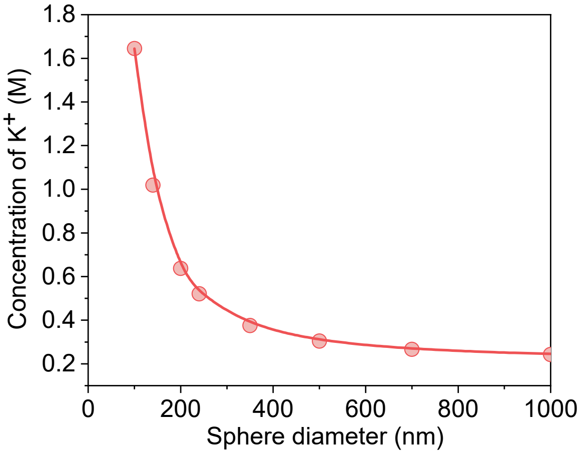

Supplementary Fig. 8 | Finite-element numerical method simulation results of the local surface K^+ concentration as a function of sphere diameter. Bulk electrolyte K^+ concentration was set at 0.2 M.

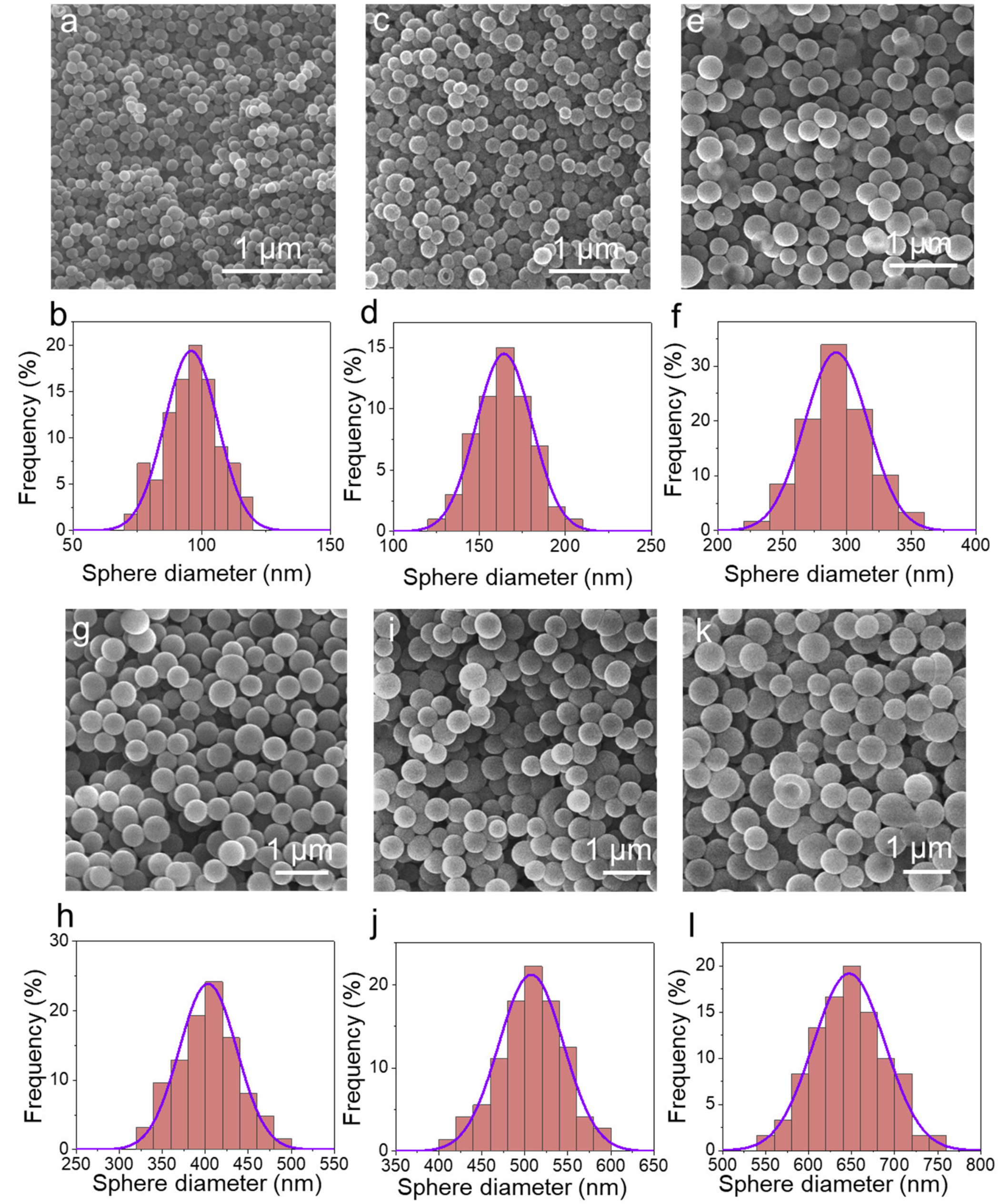

Supplementary Fig. 9 | SEM images of the polymer nanospheres with different diameters (a) 100 nm, (c)175 nm, (e) 300 nm, (g) 400 nm, (i) 500 nm and (k) 650 nm. (b), (d), (f), (h), (j) and (l) are the corresponding size distribution analysis of (a), (c), (e), (g), (i) and (k) respectively.

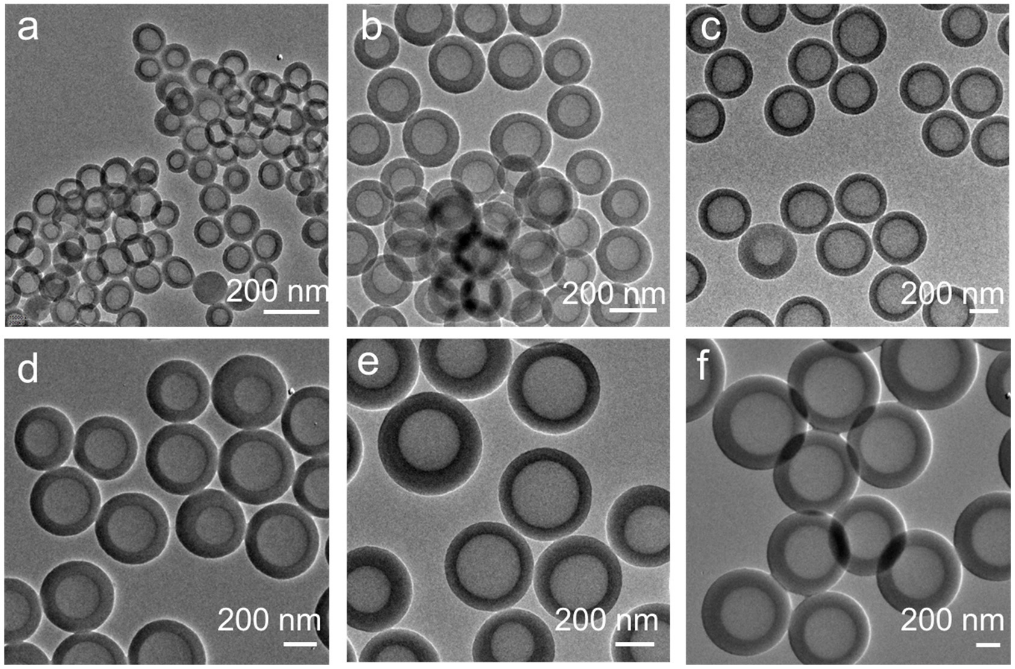

Supplementary Fig. 10 | TEM images of the polymer nanospheres with different diameters (a) 100 nm, (b)175 nm, (c) 300 nm, (d) 400 nm, (e) 500 nm and (f) 650 nm.

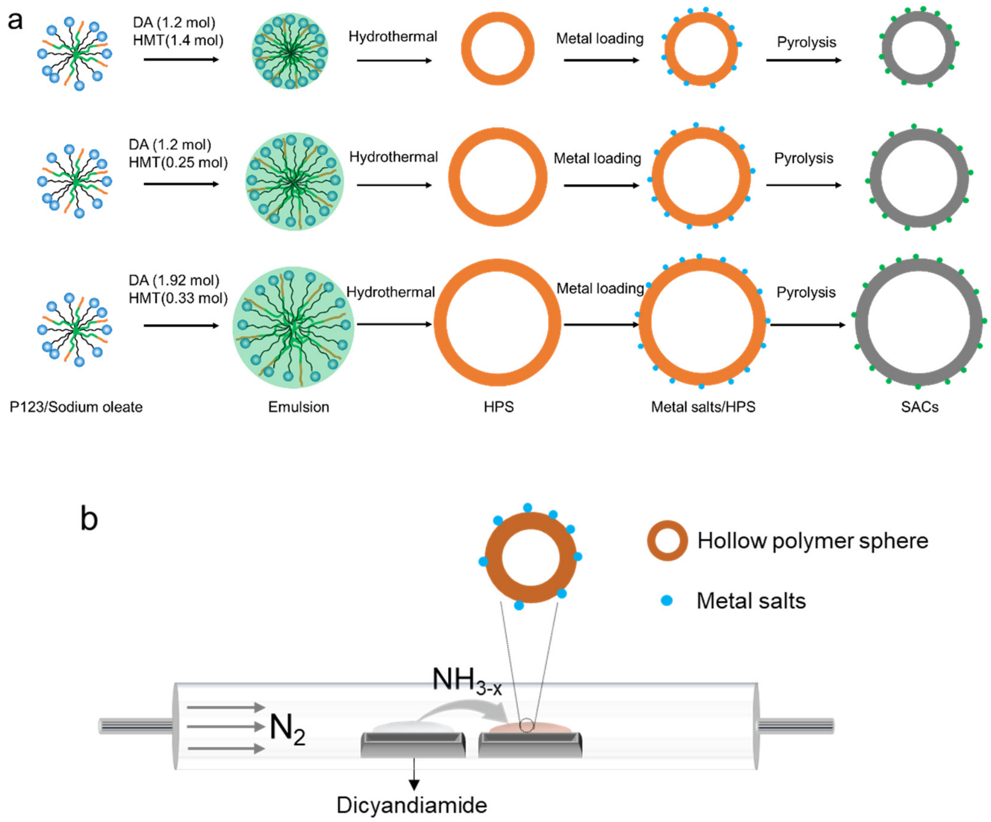

Supplementary Fig. 11 | (a) Illustration of the synthetic process for constructing M-N_4 catalysts supported on carbon nanospheres of tunable diameters. (b) Schematic showing the process involved in the pyrolysis step. See Table S1 for the synthetic parameters employed for each sphere diameter.

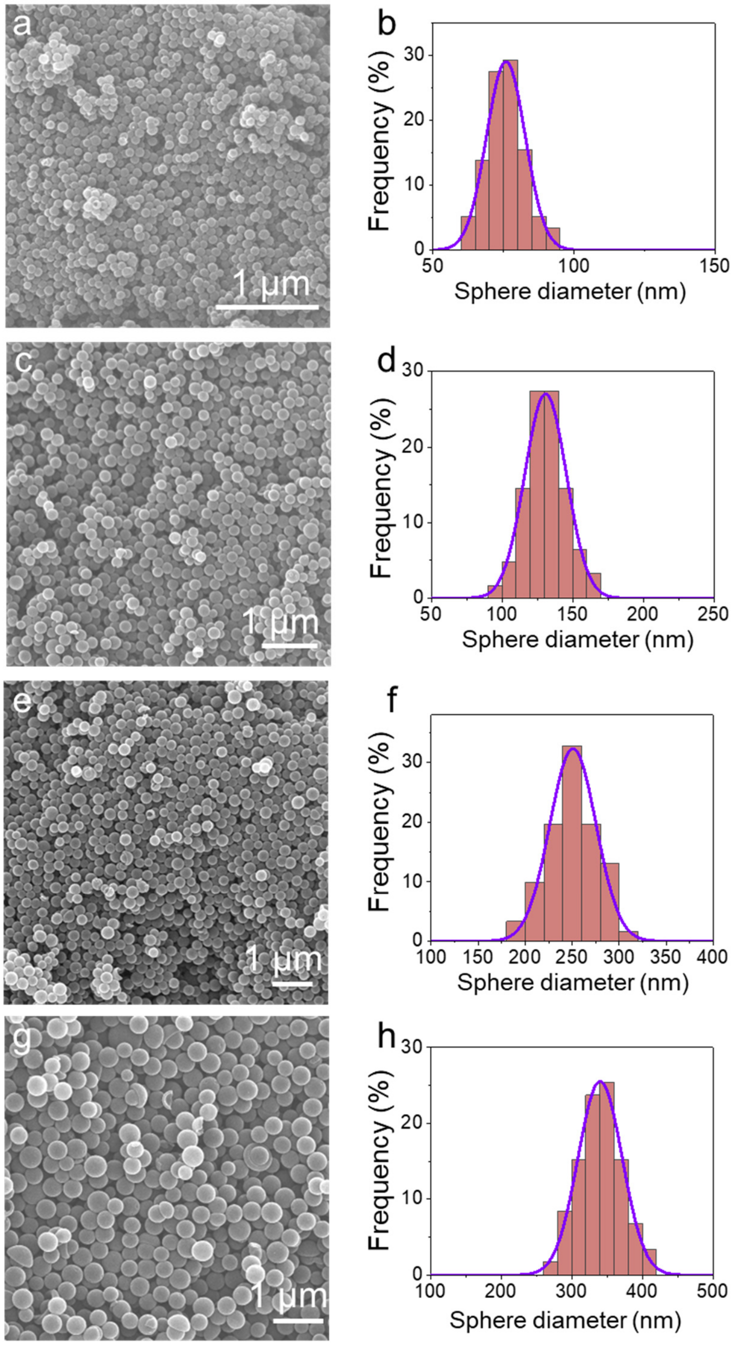

Supplementary Fig. 12 | SEM images of the Ni-SACs with different diameters: (a) 70 nm, (c) 130 nm, (e) 250 nm, (g) and 350 nm. (b), (d), (f) and (h) are the corresponding size distribution analysis of (a), (c), (e) and (g) respectively.

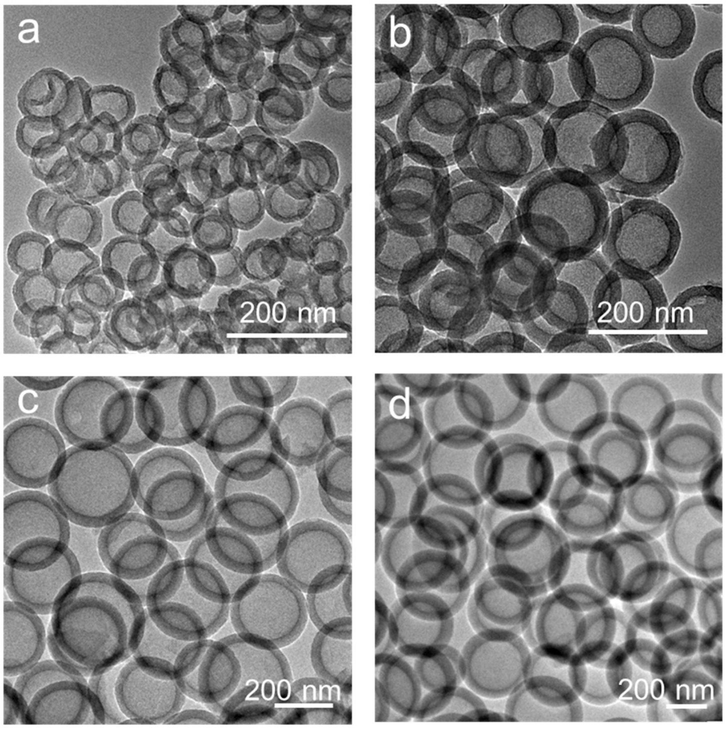

Supplementary Fig. 13 | TEM images of the Ni-SACs with different sphere diameters. (a) 70 nm, (b) 130 nm, (c) 250 nm and (d) 350 nm.

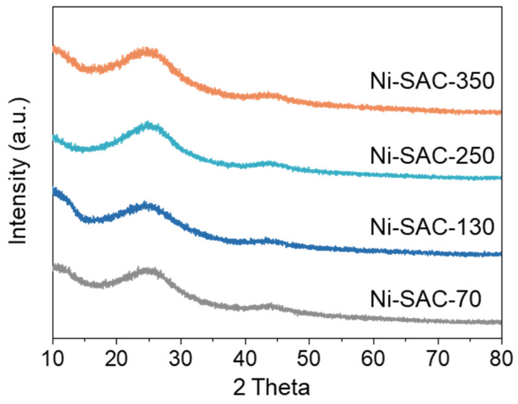

Supplementary Fig. 14 | XRD patterns of Ni-SACs with varying sphere diameters. These results indicate the absence of any metallic Ni phases, with only two broad peaks associated with graphitic carbon for all Ni-SACs.

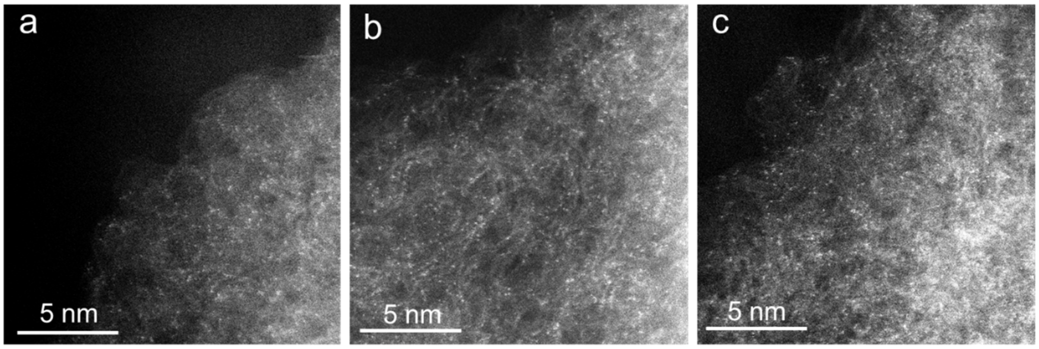

Supplementary Fig. 15 | AC-HAADF-STEM image of (a) Ni-SAC-70 nm, (b) Ni-SAC-130 nm and (c) Ni-SAC-250 nm.

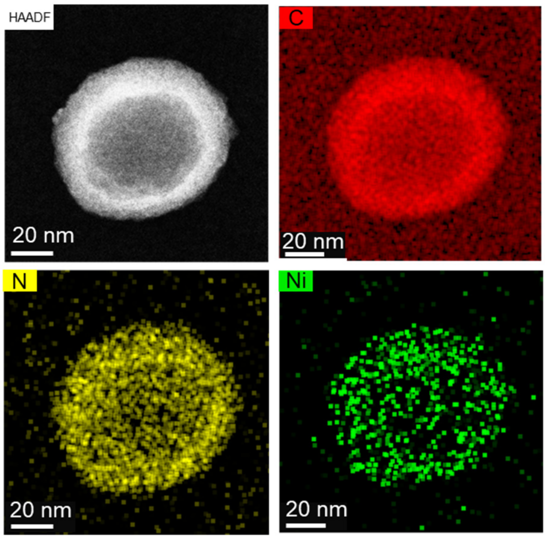

Supplementary Fig. 16 | HAADF-STEM of Ni-SAC-70 and corresponding EDS mapping images.

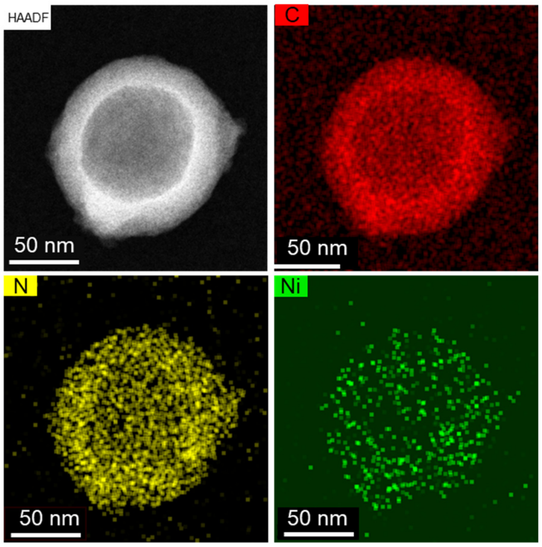

Supplementary Fig. 17 | HAADF-STEM of Ni-SAC-130 and corresponding EDS mapping images.

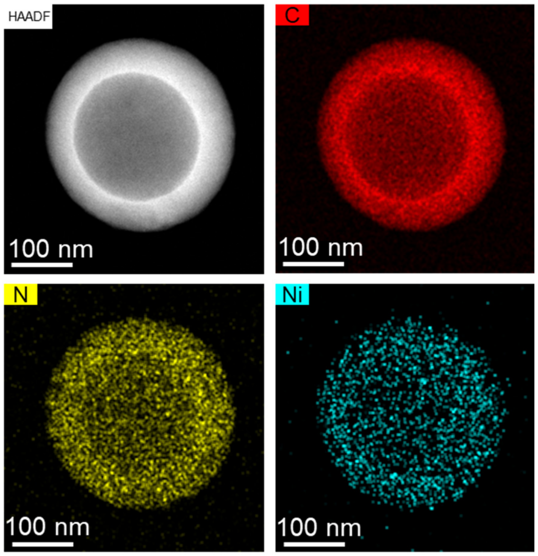

Supplementary Fig. 18 | HAADF-STEM of Ni-SAC-350 and corresponding EDS mapping images.

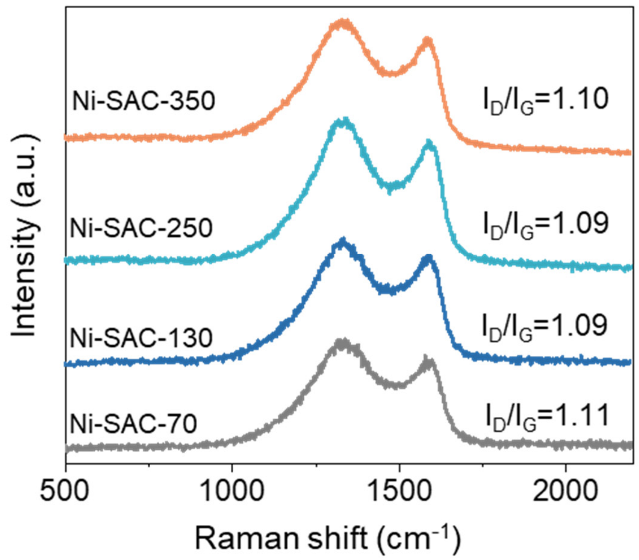

Supplementary Fig. 19 | Raman spectra of Ni-SACs with varying carbon support sphere diameter.

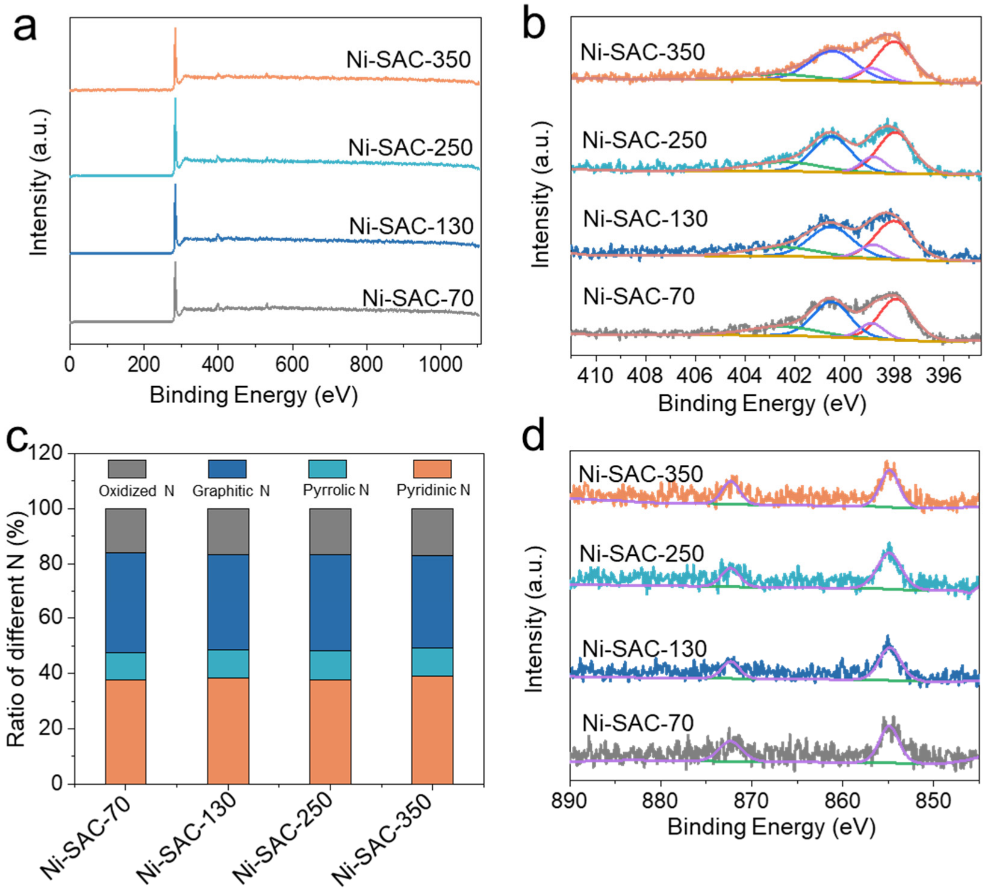

Supplementary Fig. 20 | XPS results of Ni-SACs with varying sphere diameters. (a) survey spectra, (b) XPS narrow scan spectra of the N 1*s* region, (c) calculated ratios of the different N species and (d) XPS narrow scan spectra of the Ni 2*p* region.

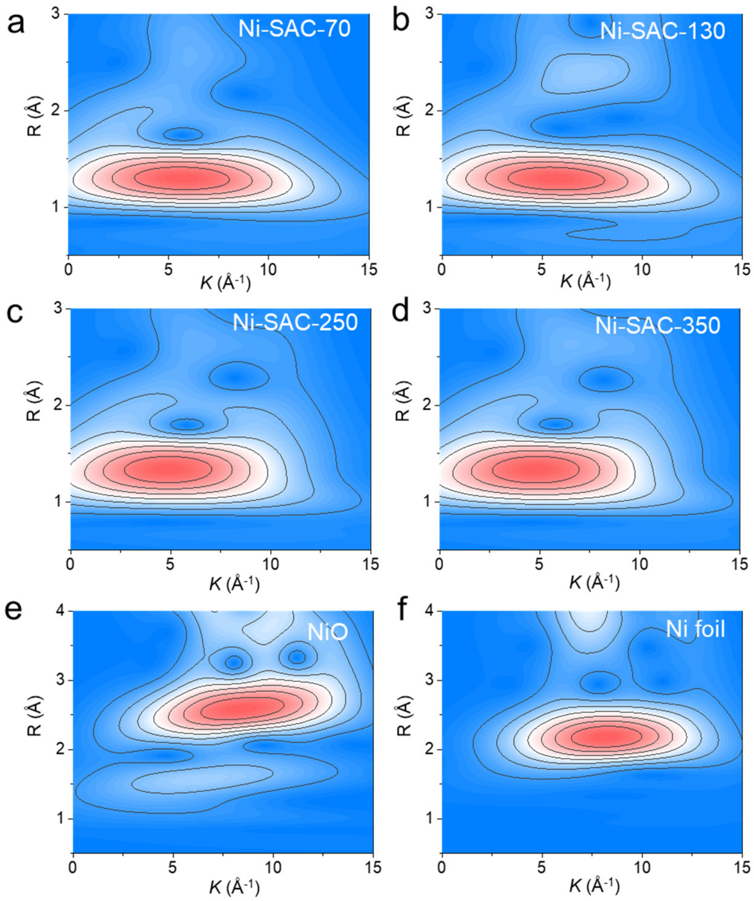

Supplementary Fig. 21 | Ni K-edge WTs of the Ni-SAC samples with different sphere diameters: (a) Ni-SAC-70, (b) Ni-SAC-130, (c) Ni-SAC-250 and (d) Ni-SAC-350. (e) and (f) are data for NiO and Ni foil respectively.

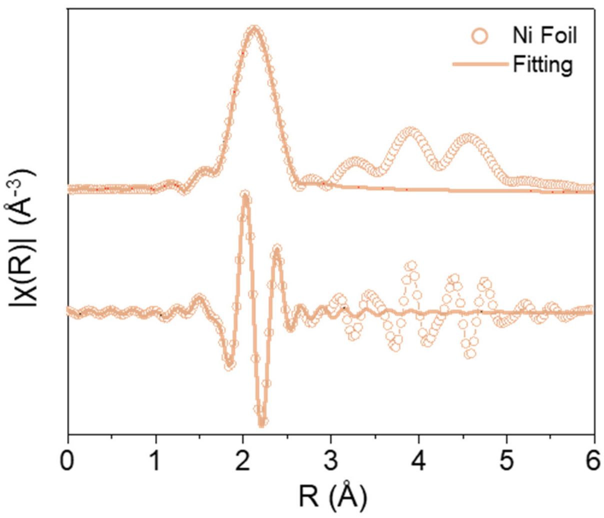

Supplementary Fig. 22 | First-shell fitting of Fourier transformations of EXAFS spectra for Ni foil. Top and bottom traces are the magnitude and imaginary part respectively.

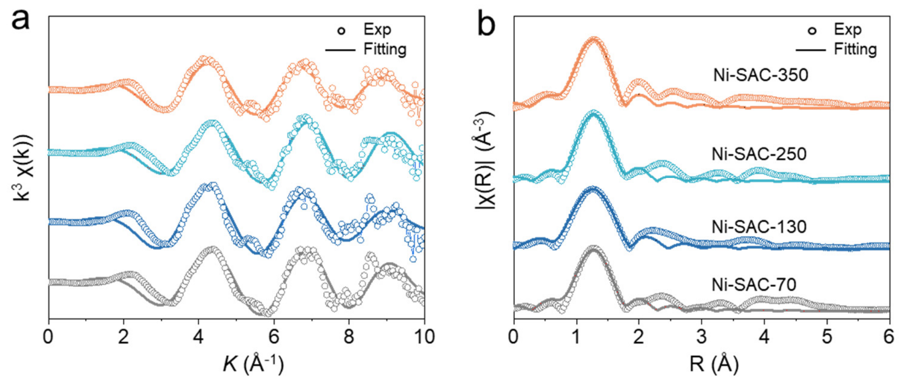

Supplementary Fig. 23 | EXAFS fitting results of the Ni K-edge of the samples with different sphere diameters at (a) k space and (b) R space.

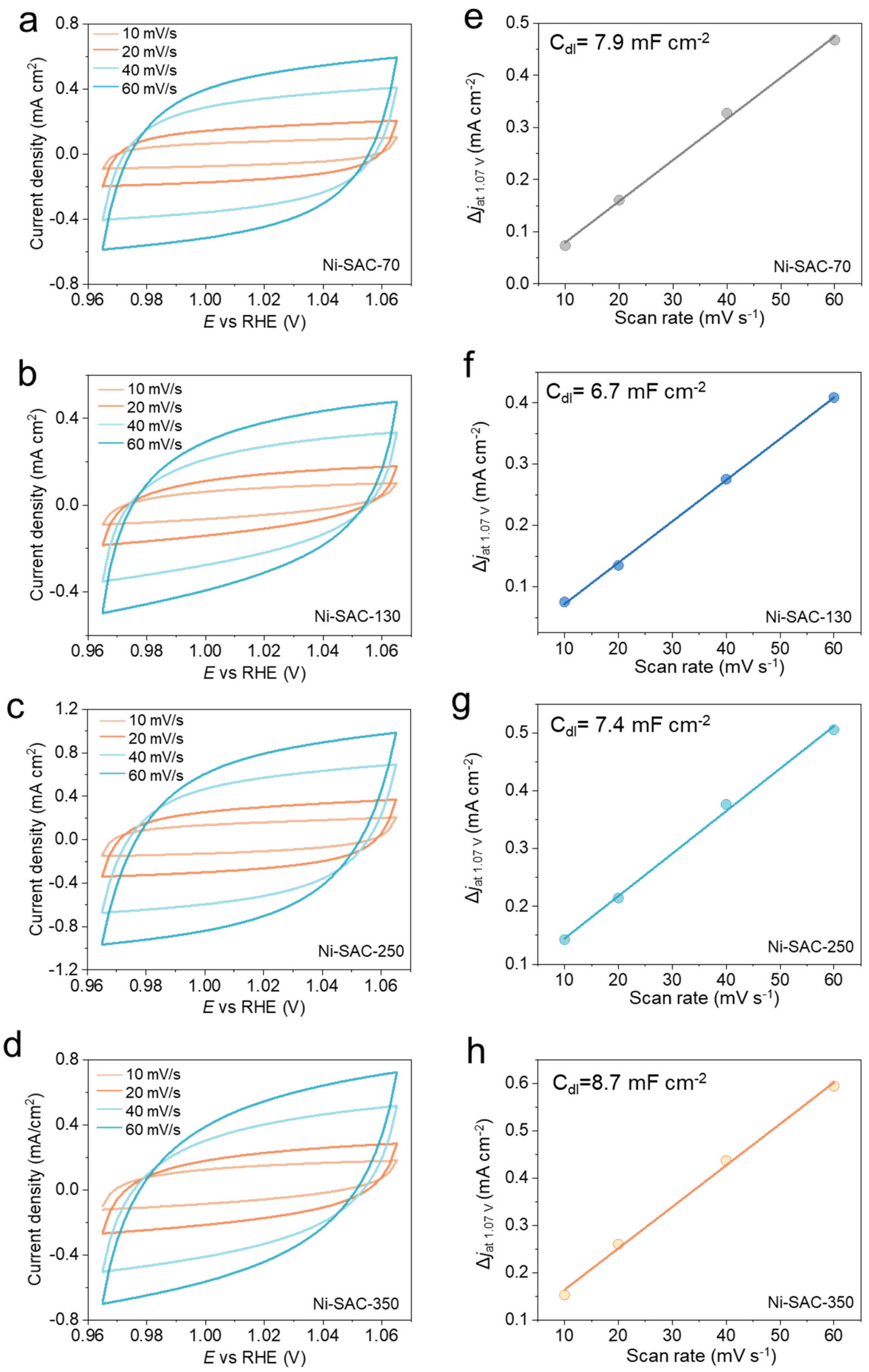

Supplementary Fig. 24 | Cyclic voltammograms for (a) Ni-SAC-70, (b) Ni-SAC-130, (c) Ni-SAC-250 and (d) Ni-SAC-350 at different scan rates from 10 to 60 mV s^{-1} respectively. (e-h) are corresponding plots of the capacitive current vs scan rate, with the calculated capacitance shown for each case.

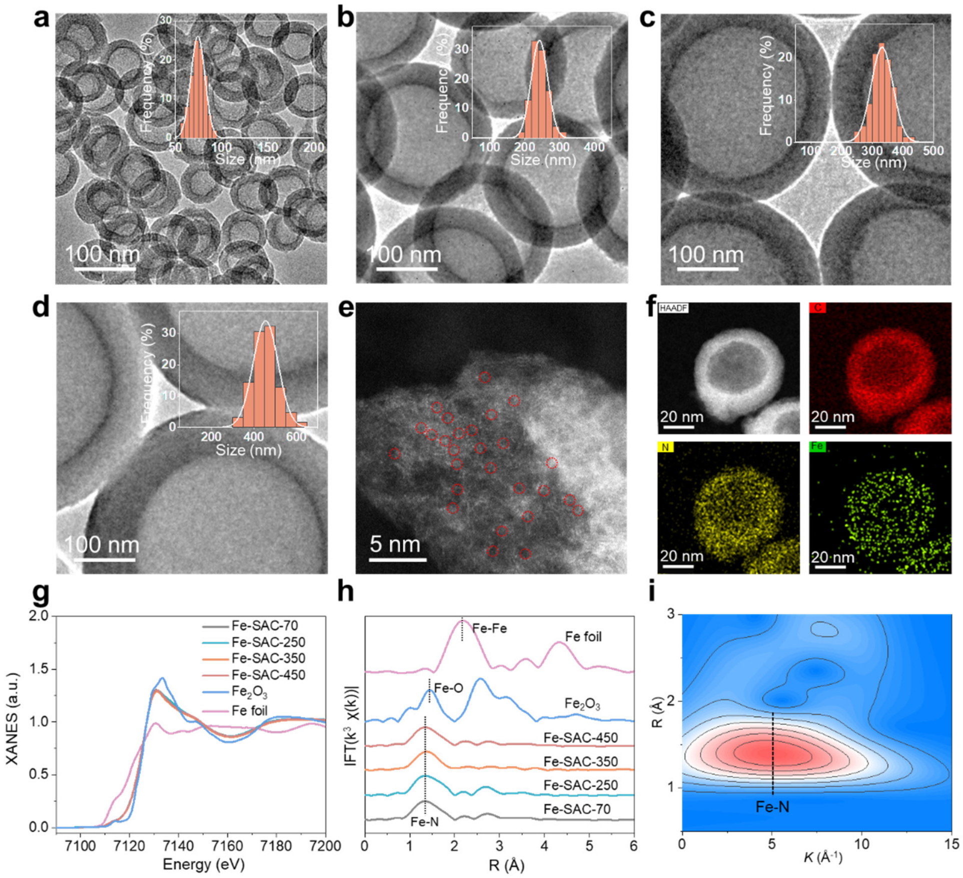

Supplementary Fig. 25 | (a) to (d) TEM images of Fe-SACs, which consist of Fe single-atoms supported on hollow carbon nanospheres of varying diameter. (a) Fe-SAC-70, (b) Fe-SAC-250, (c) Fe-SAC-350 and (d) Fe-SAC-450. The inset of each image shows the size distribution of the carbon nanospheres. (e) AC-HAADF-STEM image of Fe-SAC-70. (f) HAADF-STEM and corresponding EDS mapping images of Fe-SAC-70. (g) Fe K-edge XANES spectra and (h) Fe K-edge Fourier-transformed (FT) k^3-weighted $\chi(k)$ functions of Fe-SACs with different sphere diameters. (i) Fe K-edge WTs of Fe-SAC-70.

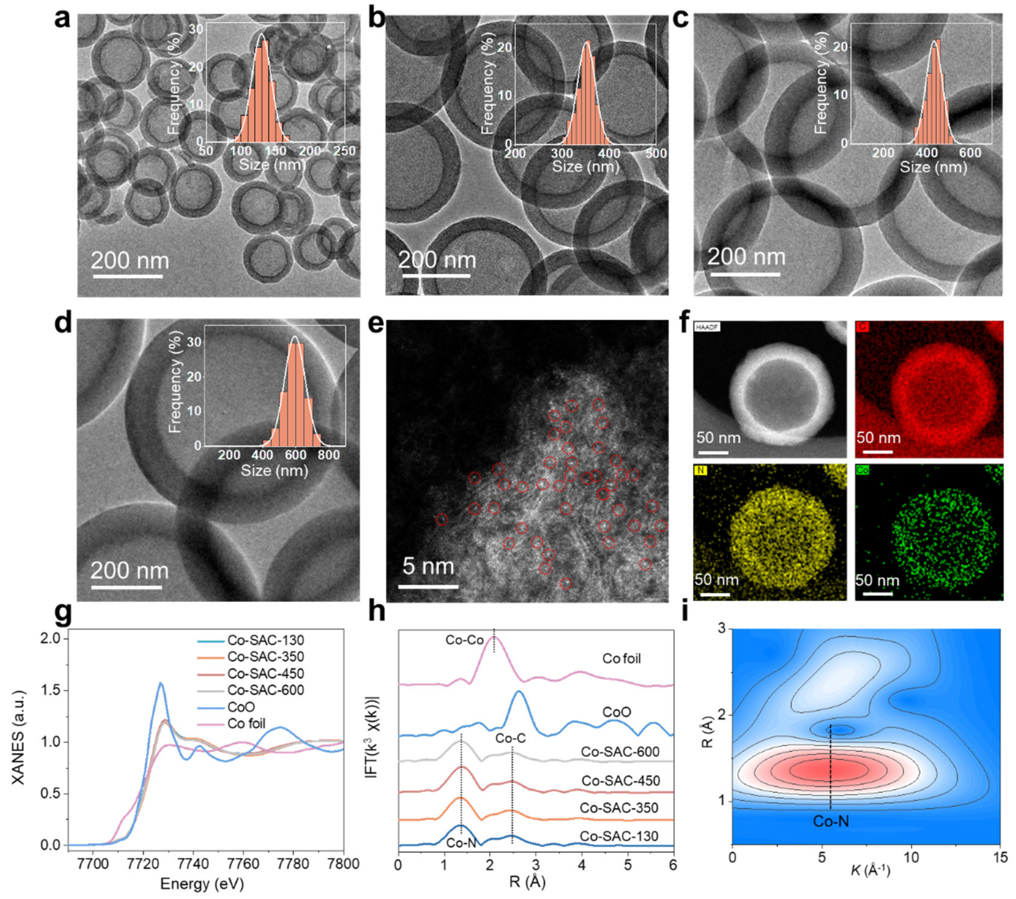

Supplementary Fig. 26 | (a) to (d) TEM images of Co-SACs, which consist of Co single-atoms supported on hollow carbon nanospheres of varying diameter. (a) Co-SAC-130, (b) Co-SAC-350, (c) Co-SAC-450 and (d) Co-SAC-600. The inset of each image shows the size distribution of the carbon nanospheres. (e) AC-HAADF-STEM image of Co-SAC-130. (f) HAADF-STEM and corresponding EDS mapping images of Co-SAC-130. (g) Co K-edge XANES spectra and (h) Co K-edge Fourier-transformed (FT) k^3-weighted χ(k) functions of Co-SACs with different sphere diameters. (i) Co K-edge WTs of Co-SAC-130.

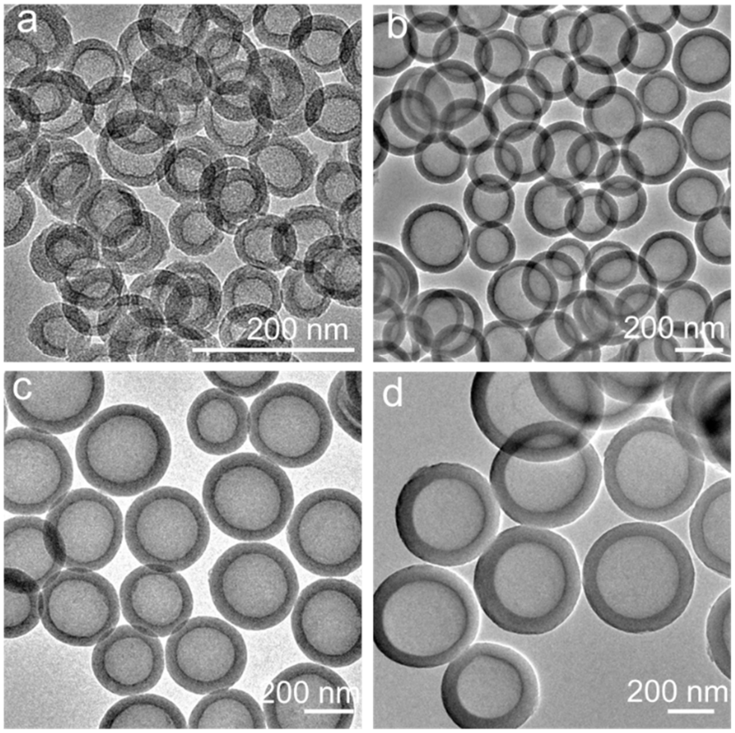

Supplementary Fig. 27 | Large area TEM images of the Fe-SACs with different carbon support sphere diameters. (a) 70 nm, (b) 250 nm, (c) 350 nm and (d) 450 nm.

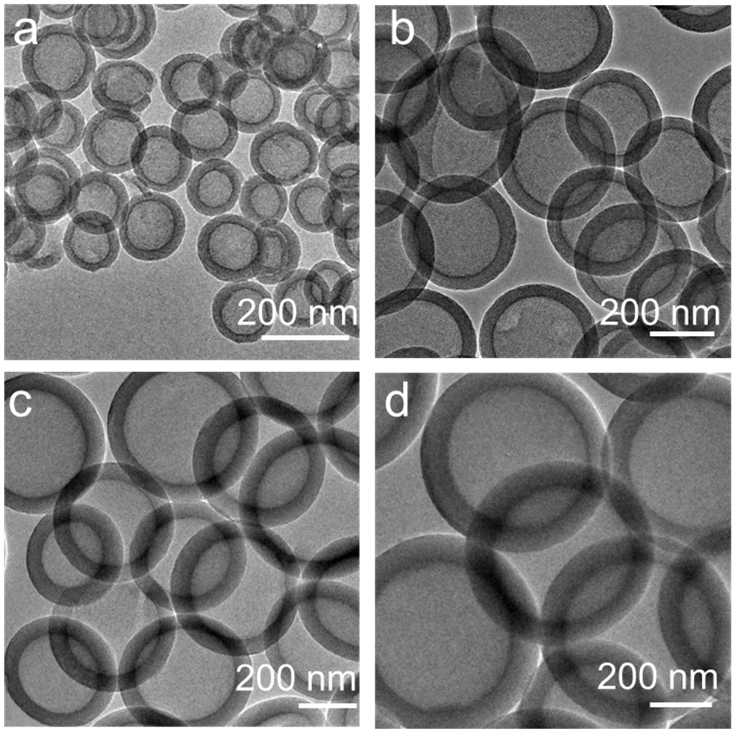

Supplementary Fig. 28 | TEM images of the Co-SACs with different carbon support sphere diameters. (a) 130 nm, (b) 350 nm, (c) 450 nm and (d) 600 nm.

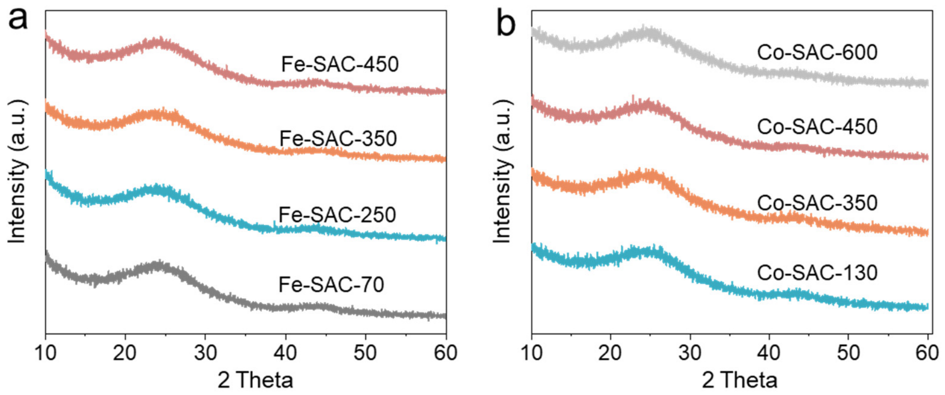

Supplementary Fig. 29 | XRD patterns of Fe-SACs (a) and Co-SACs (b) with different sphere diameters. These results indicate the absence of any metallic Fe and Co phases, with only two broad peaks associated with graphitic carbon for all Fe-SACs and Co-SACs.

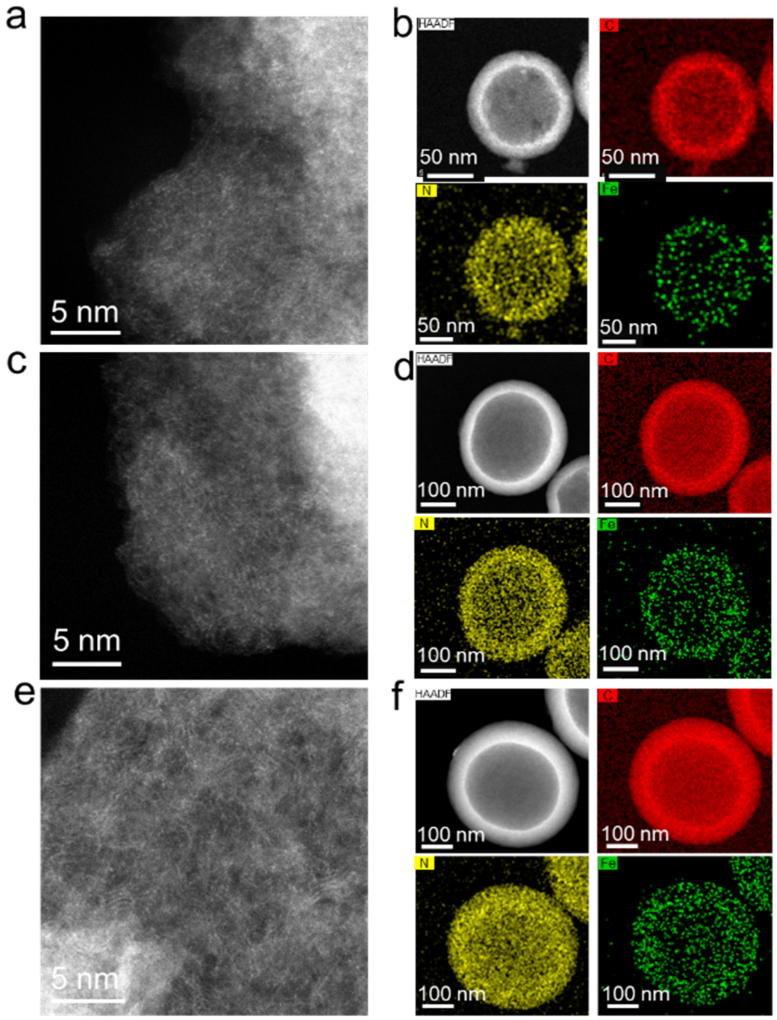

Supplementary Fig. 30 | HAADF-STEM images of Fe-SACs with different sphere diameters: (a) 250 nm, (c) 350 nm and (e) 450 nm. Corresponding EDS mapping images are shown in (b), (d) and (f) respectively.

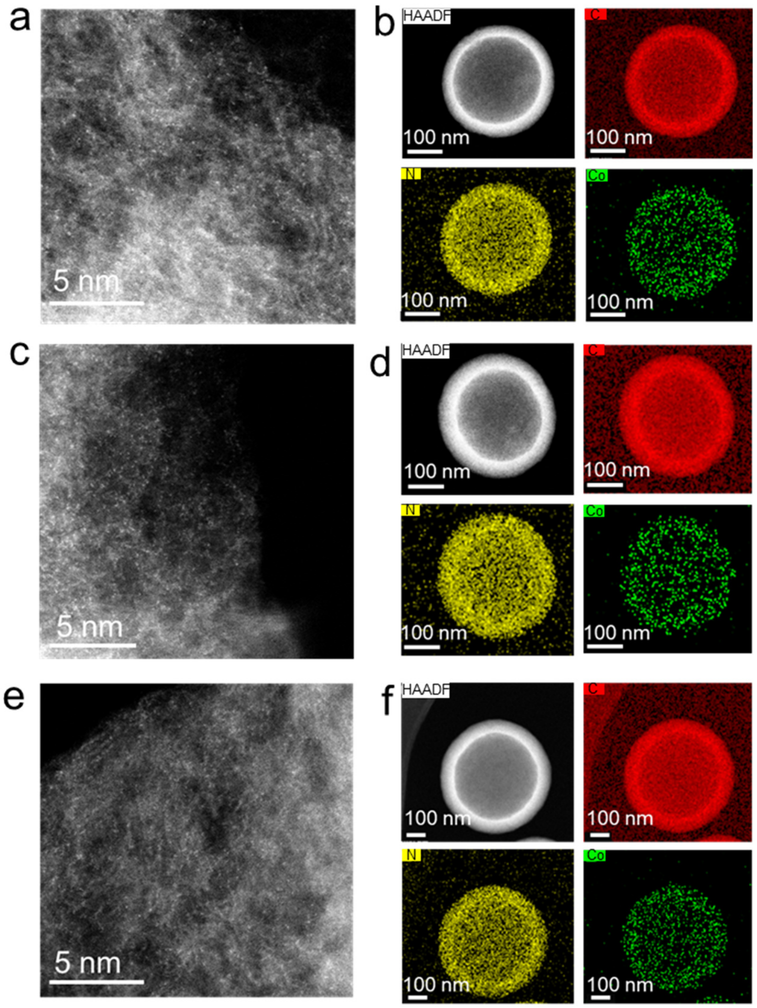

Supplementary Fig. 31 | HAADF-STEM images of Co-SACs with different sphere diameters: (a) 350 nm, (c) 450 nm and (e) 600 nm. corresponding EDS mapping images are shown in (b), (d) and (f) respectively.

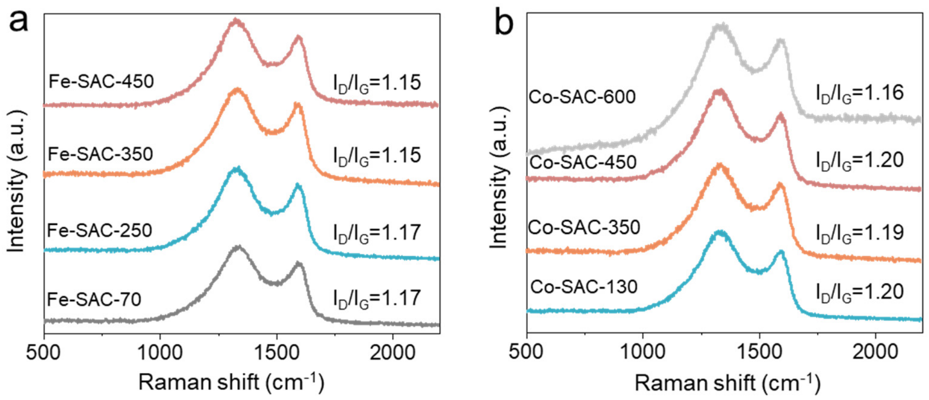

Supplementary Fig. 32 | Raman spectra of (a) Fe-SACs and (b) Co-SACs with different sphere diameters. These results exhibit similar D/G ratios of around 1.15 for the Fe-SACs and 1.20 for Co-SACs, which indicates that they all have similar degrees of graphitization.

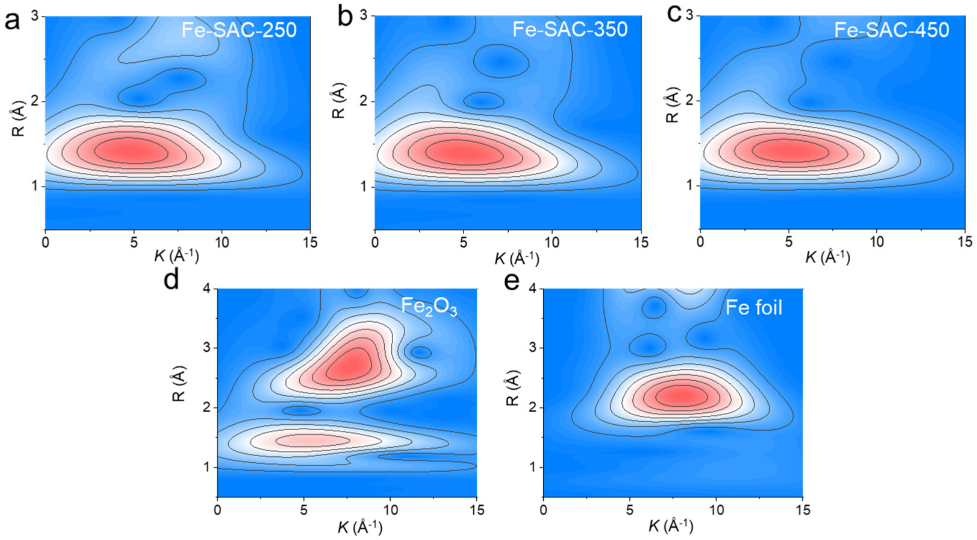

Supplementary Fig. 33 | Fe K-edge WTs of the Fe-SACs with different sphere diameters: (a) Fe-SAC-250, (b) Fe-SAC-350 and (c) Fe-SAC-450. (d) and (e) are data for Fe_2O_3 and Fe foil respectively.

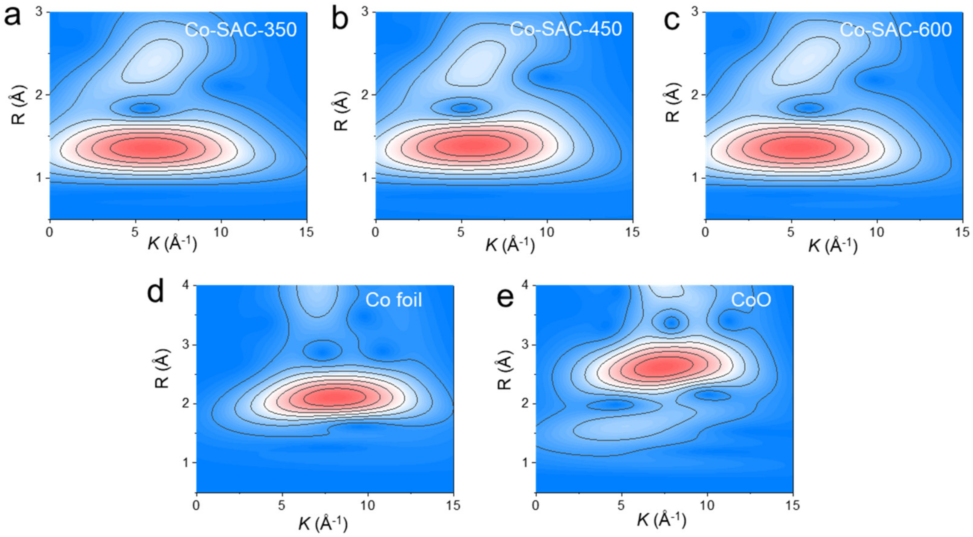

Supplementary Fig. 34 | Co K-edge WTs of the Co-SACs with different sphere diameters: (a) Co-SAC-350, (b) Co-SAC-450 and (c) Co-SAC-600. (d) and (e) are data for Co foil and CoO respectively.

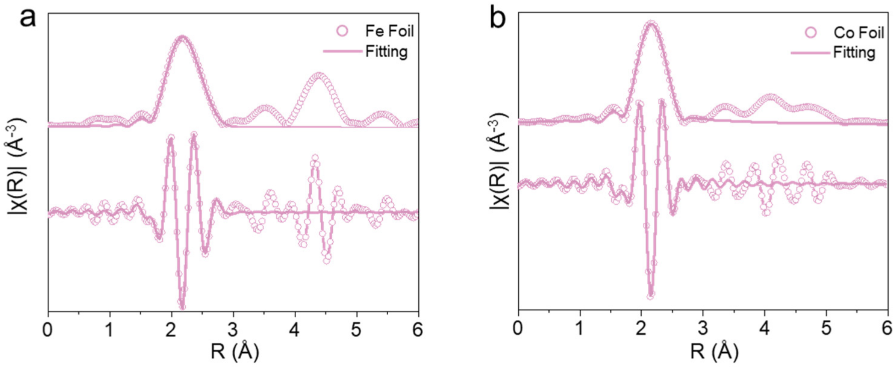

Supplementary Fig. 35 | First-shell fitting of Fourier transformations of EXAFS spectra for (a) Fe foil and (b) Co foil. Top and bottom traces are the magnitude and imaginary part respectively.

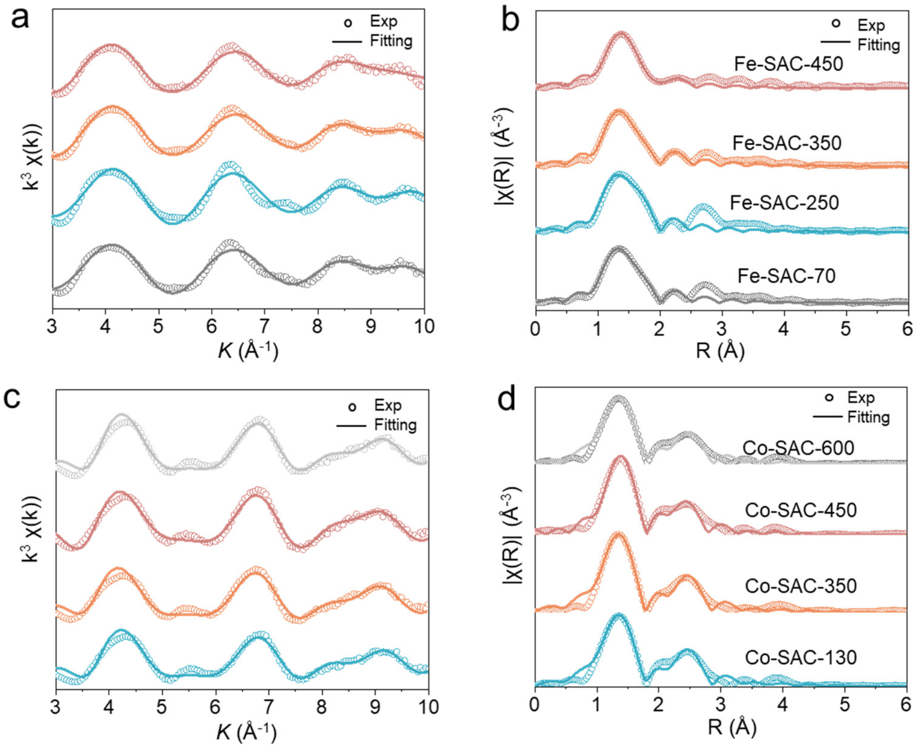

Supplementary Fig. 36 | EXAFS fitting results of Fe K-edge of the Fe-SACs with different sphere diameters at (a) k space and (b) R space. EXAFS fitting results of Co K-edge of the Co-SACs with different sphere diameters at (c) k space and (d) R space.

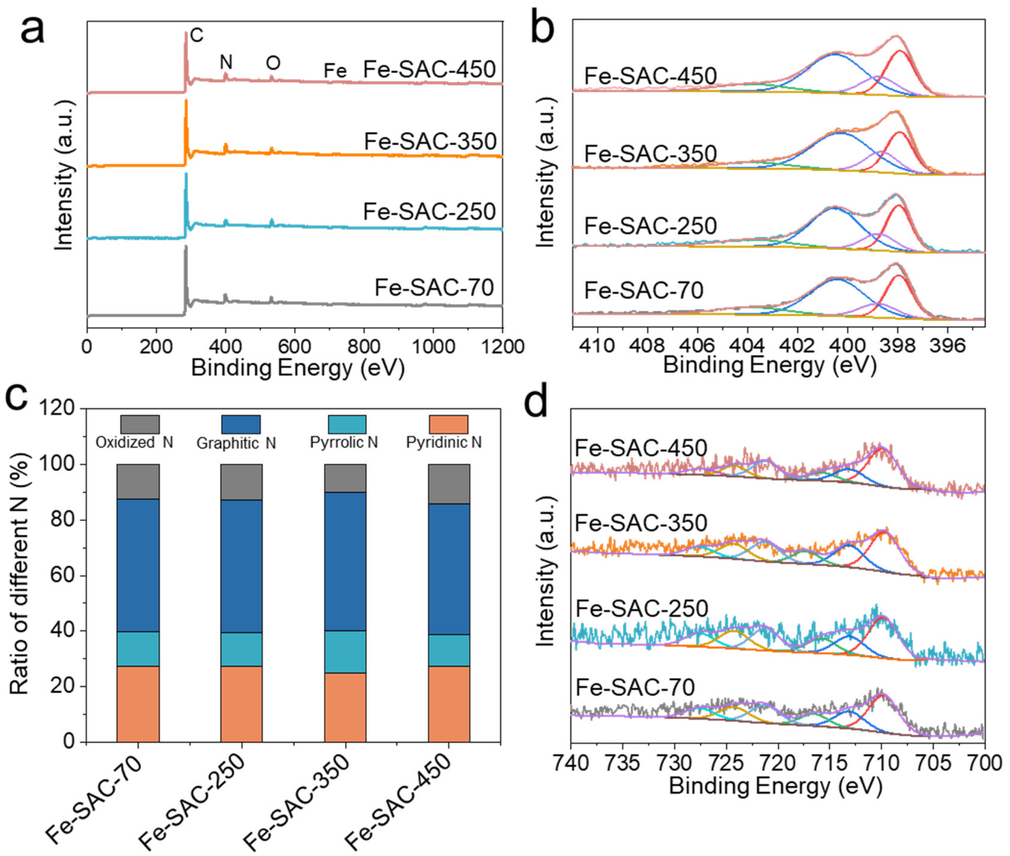

Supplementary Fig. 37 | XPS results for Fe-SACs of varying sphere diameter. (a) survey spectra, (b) XPS narrow scan spectra for the N 1*s* region, (c) ratios of the different N species and (d) XPS narrow scan spectra for the Fe 2*p* region. The N 1 s spectrum reveals the existence of pyridinic N (~397.9 eV), pyrrolic N (~398.7 eV), graphitic N (~400.4 eV) and oxidized N (~403.3 eV) for all the Fe-SACs. We find that the ratio of the different N species is similar for all SACs, regardless of the sphere diameter. Notably, the Fe 2*p* spectrum of Fe-SAC shows the positively charged iron species without obvious zero-valent iron (~706.7 eV)[1], indicating that Fe atoms are coordinated by the substrate N/C atoms, and is consistent with the XANES results.

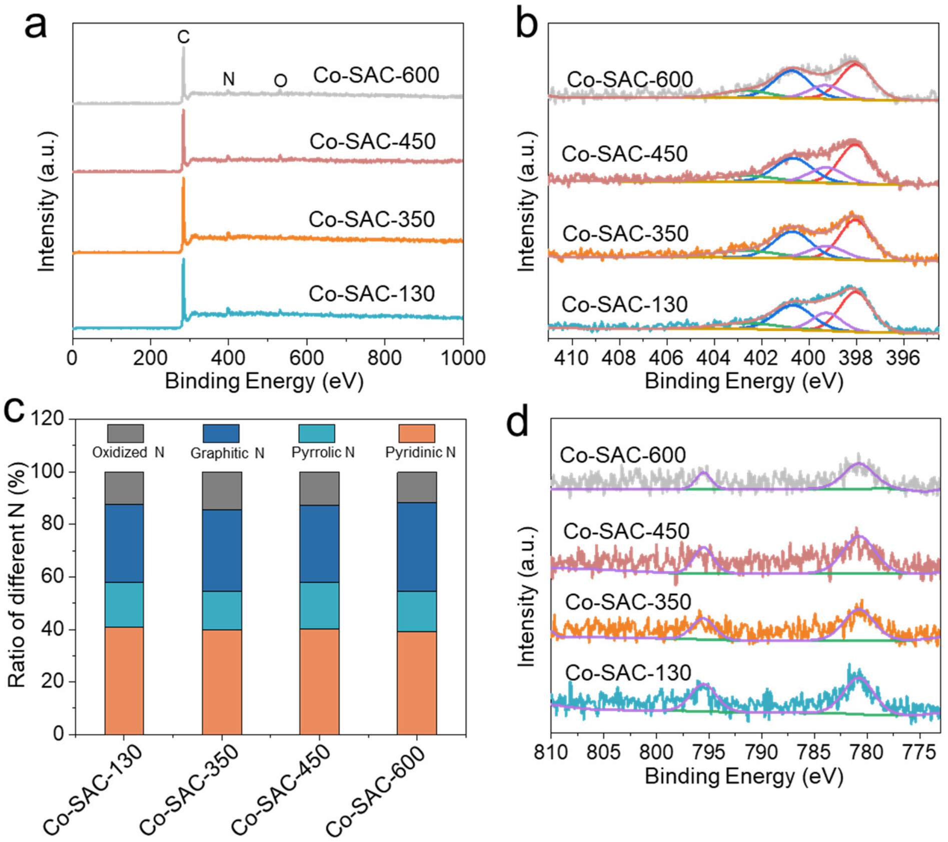

Supplementary Fig. 38 | XPS results for Co-SACs of varying sphere diameter. (a) survey spectra, (b) XPS narrow scan spectra of the N 1*s* region, (c) ratios of the different N species and (d) XPS narrow scan spectra of the Co 2*p* region. The N 1 s spectrum reveals the existence of pyridinic N (~398.0 eV), pyrrolic N (~399.3 eV), graphitic N (~400.6 eV) and oxidized N (~402.7 eV) for all the Co-SACs and the ratio of the different N species is similar for all SACs, regardless of the sphere diameter. The binding energy of Co 2*p* in the Co SACs are all located at ~780.8 eV. This indicates a higher oxidation state compared to metallic Co (778.1 eV) but is lower than that of Co^{3+} (781.0 eV)[2].

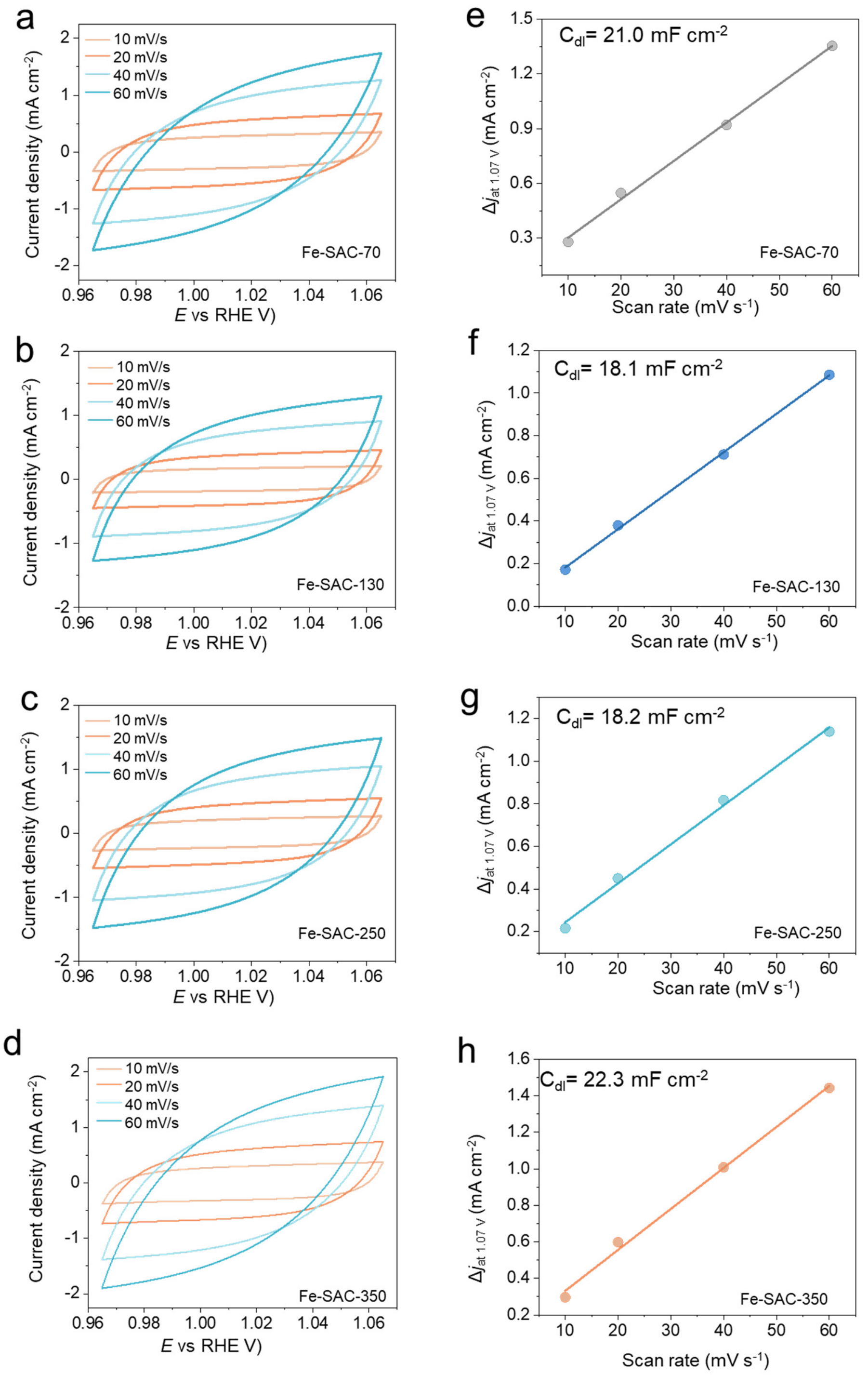

Supplementary Fig. 39 | Cyclic voltammograms for (a) Fe-SAC-70, (b) Fe-SAC-130, (c) Fe-SAC-250 and (d) Fe-SAC-350 at different scan rates from 10 to 60 mV s^{-1} respectively. (e-h) are plots of the capacitive current vs scan rate, with the corresponding calculated double-layer capacitance shown in each case.

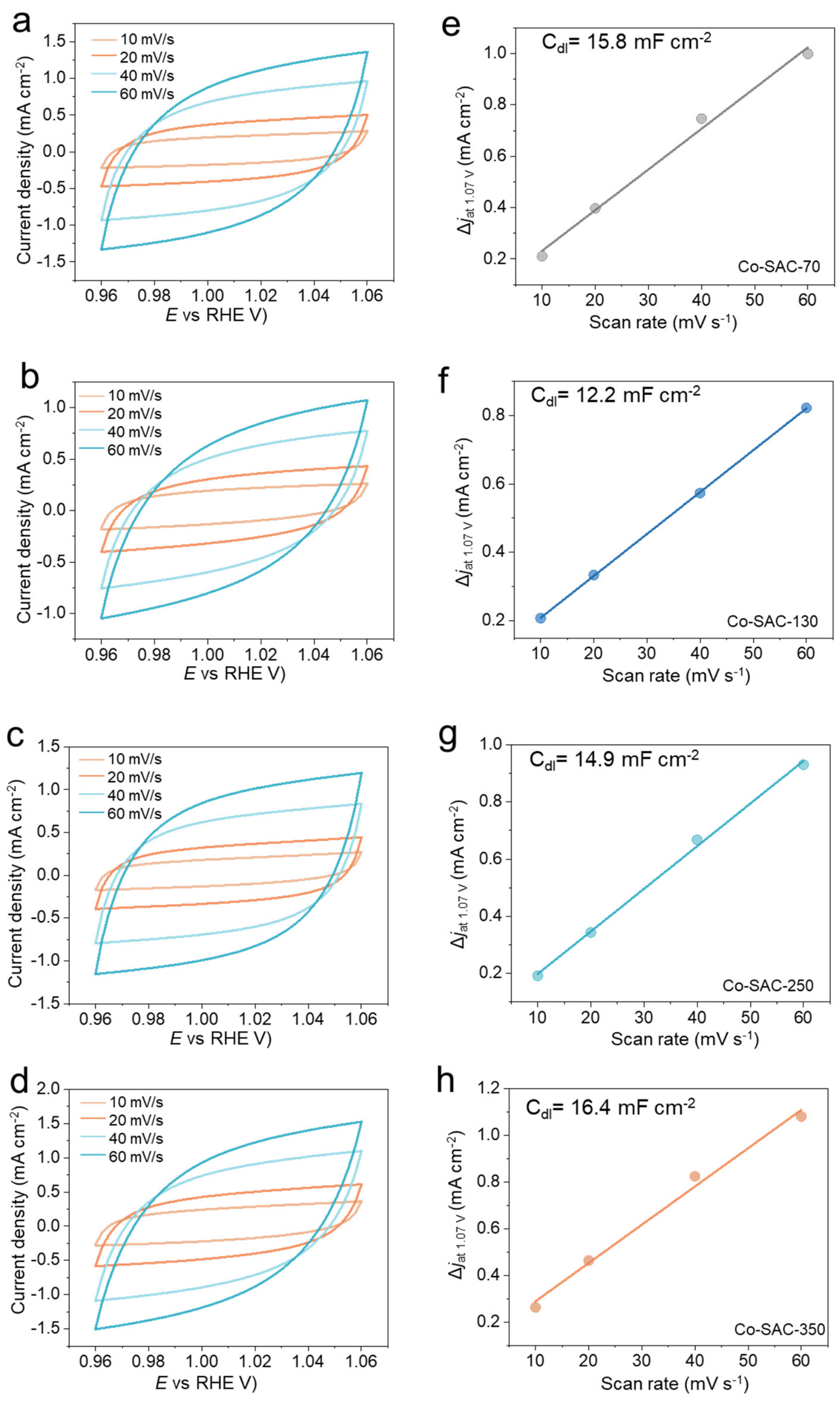

Supplementary Fig. 40 | Cyclic voltammograms for (a) Co-SAC-70, (b) Co-SAC-130, (c) Co-SAC-250 and (d) Co-SAC-350 at different scan rates from 10 to 60 mV s^{-1} respectively. (e-h) are plots of the capacitive current vs scan rate, with the corresponding calculated double-layer capacitance shown in each case.

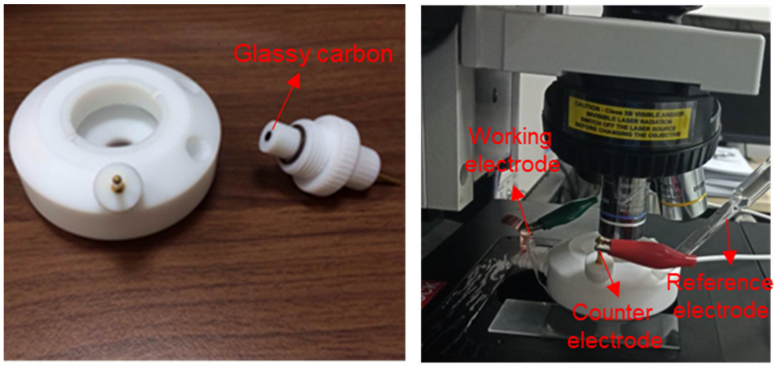

Supplementary Fig. 41 | Photograph of the custom-built electrochemical cell used for *in-situ* Raman spectroscopy measurements.

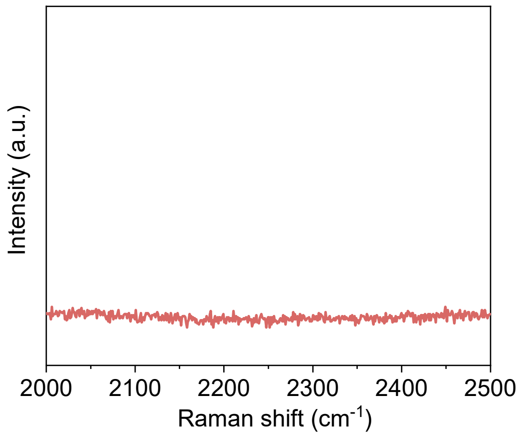

Supplementary Fig. 42 | SHINERS enhanced Raman spectrum of Ni-SAC-250 without adsorbed SCN^- as a control.

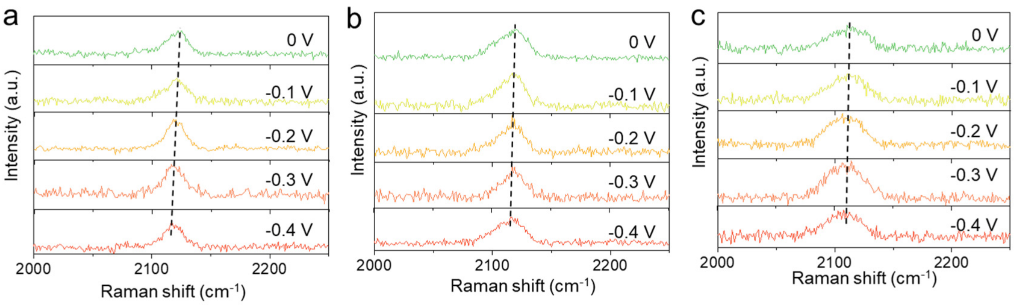

Supplementary Fig. 43 | Potential-dependent SHINERS spectra of SCN^- adsorbed on (a) Ni-SAC-130, (b) Ni-SAC-250 and (c) Ni-SAC-350 in 0.1 M $NaClO_4$ solution.

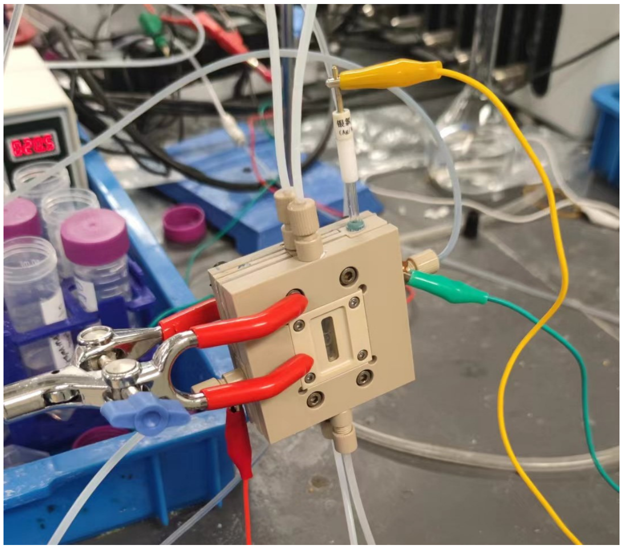

Supplementary Fig. 44 | Photograph of the custom-built electrochemical cell used for the CO_2R measurements.

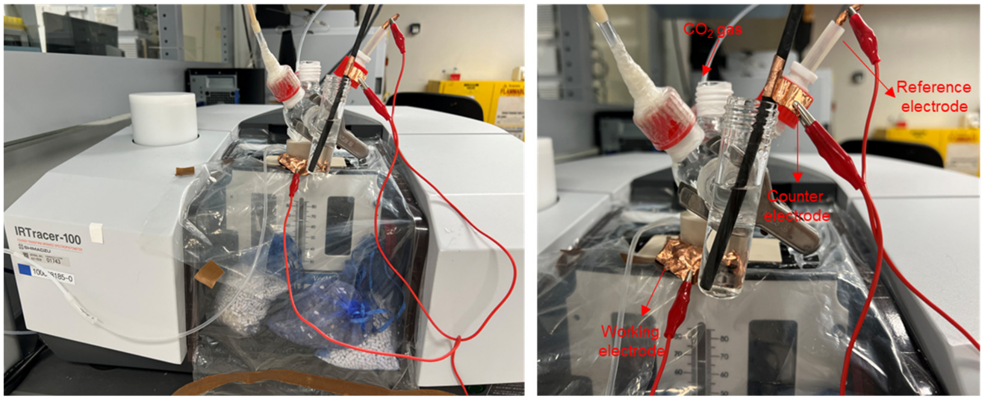

Supplementary Fig. 45 | Photograph of the custom-built electrochemical cell used for the *in-situ* ATR-IR measurements.

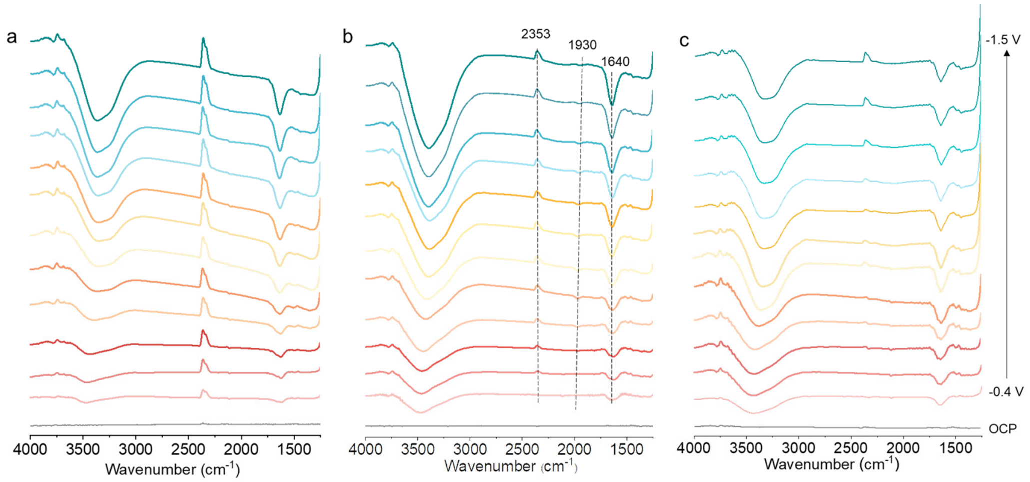

Supplementary Fig. 46 | *In situ* FTIR spectra of Ni-SACs with different sphere diameter measured at E = −0.4 V to −1.5 V vs. Ag/AgCl in pH 1 acidic electrolyte (0.1 M K_2SO4 + H_2SO_4). (a) Ni-SAC-130, (b) Ni-SAC-250 and (c) Ni-SAC-350. Peaks located at around 2353 cm^{-1} and 1640 cm^{-1} can be attributed to CO_2 absorption and stretching vibration of H_2O, respectively. The peak at 1930 cm^{-1} is a characteristic peak belonging to the stretching of a single metal site adsorbed CO[3,4]. The accumulated metal-adsorbed CO could be detected over Ni-SAC-250, but was not observed from the Ni-SAC-130 and Ni-SAC-350 samples, indicating that Ni-SAC-250 is better at converting CO_2 to CO[3,5]. In addition, no apparent peak signal belonging to *COOH was observed in the spectrum of the three investigated catalysts. This might be attributed to the fact that acidic electrolyte was employed. This is consistent with a previously reported result, where *COOH was detected in neutral electrolyte but could not be detected in acidic electrolyte over the same catalyst[6].

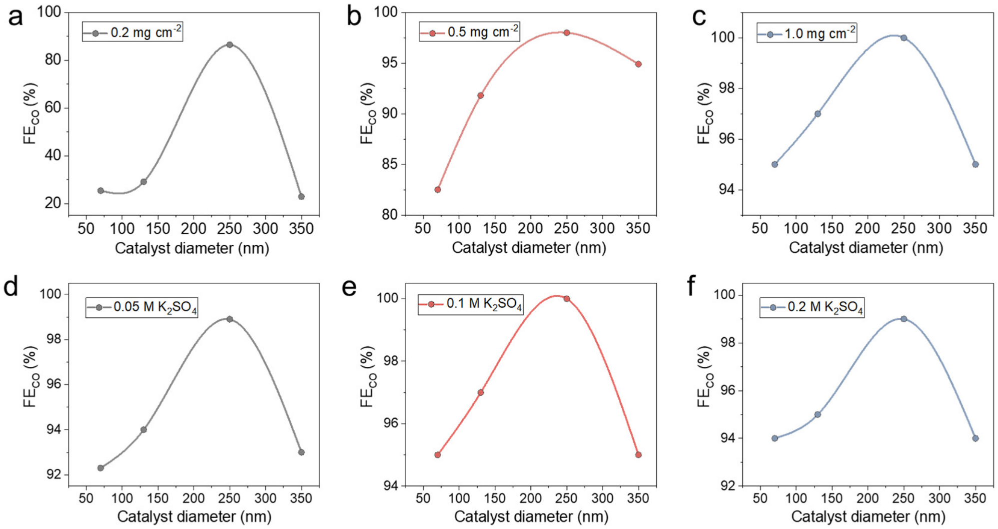

Supplementary Fig. 47 | FE towards CO for Ni-SACs with different sphere diameters under various conditions. (a-c) Different catalyst loadings (0.1M K_2SO_4 pH=1 H_2SO_4 solution) and (d-f) different concentration of K_2SO_4 electrolyte (pH=1 H_2SO_4 solution) at -1.5 V vs RHE.

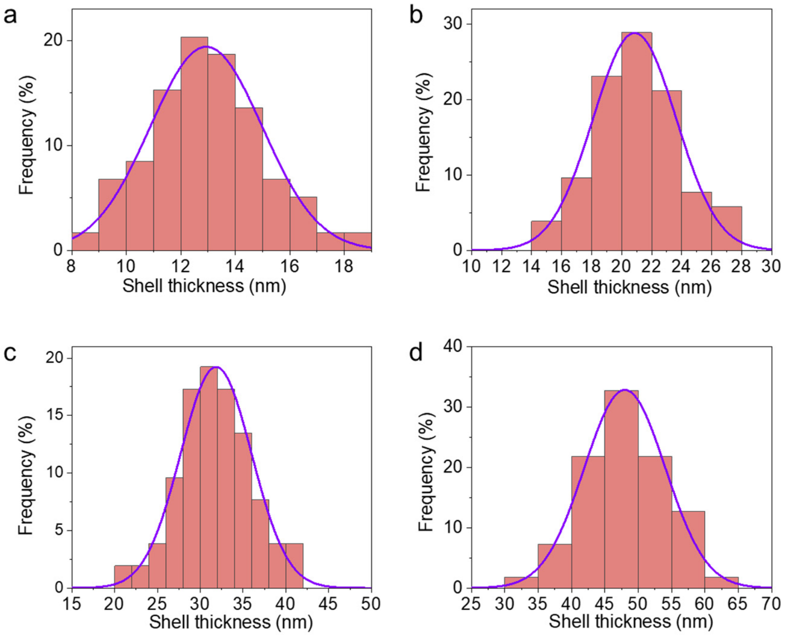

Supplementary Fig. 48 | Shell thickness analysis of Ni-SACs catalysts based on TEM images (a) Ni-SAC-70, (b) Ni-SAC-130, (c) Ni-SAC-250 and (d) Ni-SAC-350.

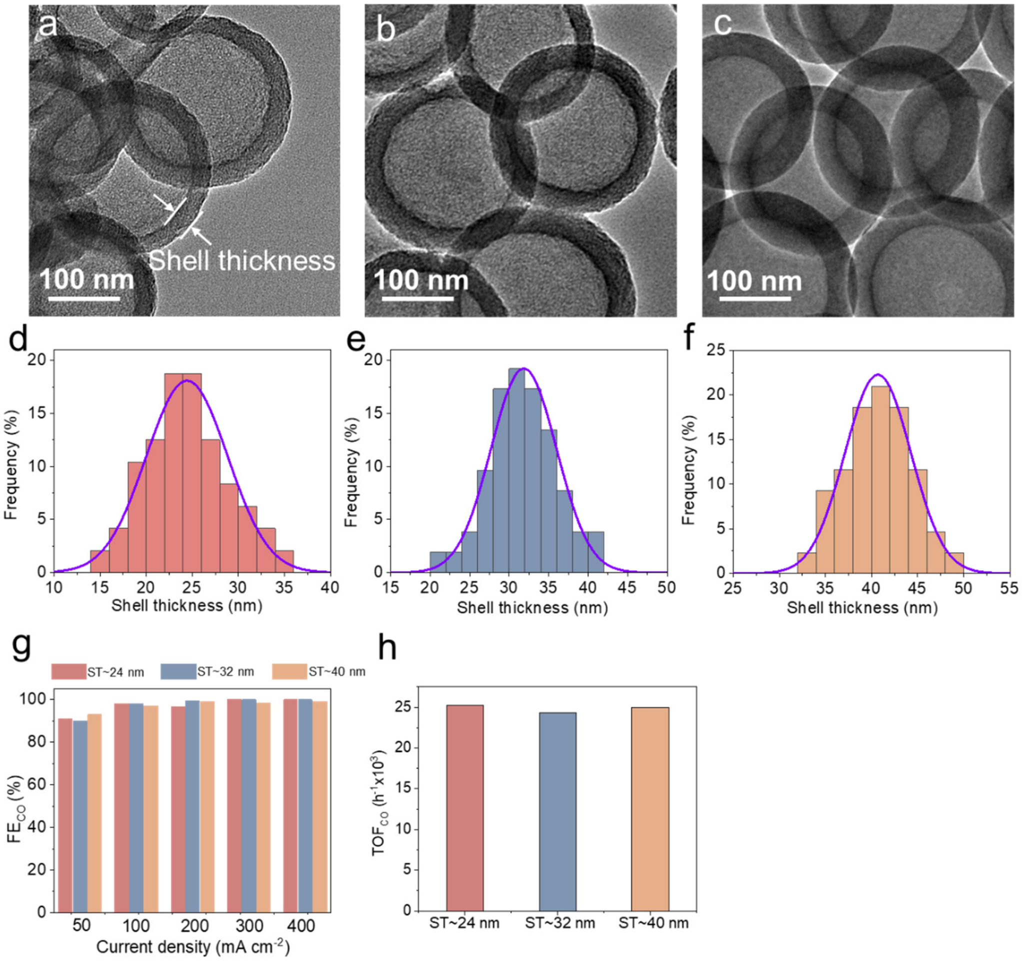

Supplementary Fig. 49 | (a-c) TEM image of Ni-SACs with similar diameter (250 nm) but different shell thicknesses and (d-f) their corresponding shell thickness analysis. (g) FE for CO production under different current densities and (h) TOFs at -1.5 V vs. RHE over the three catalysts with different shell thicknesses. ST stands for shell thickness.

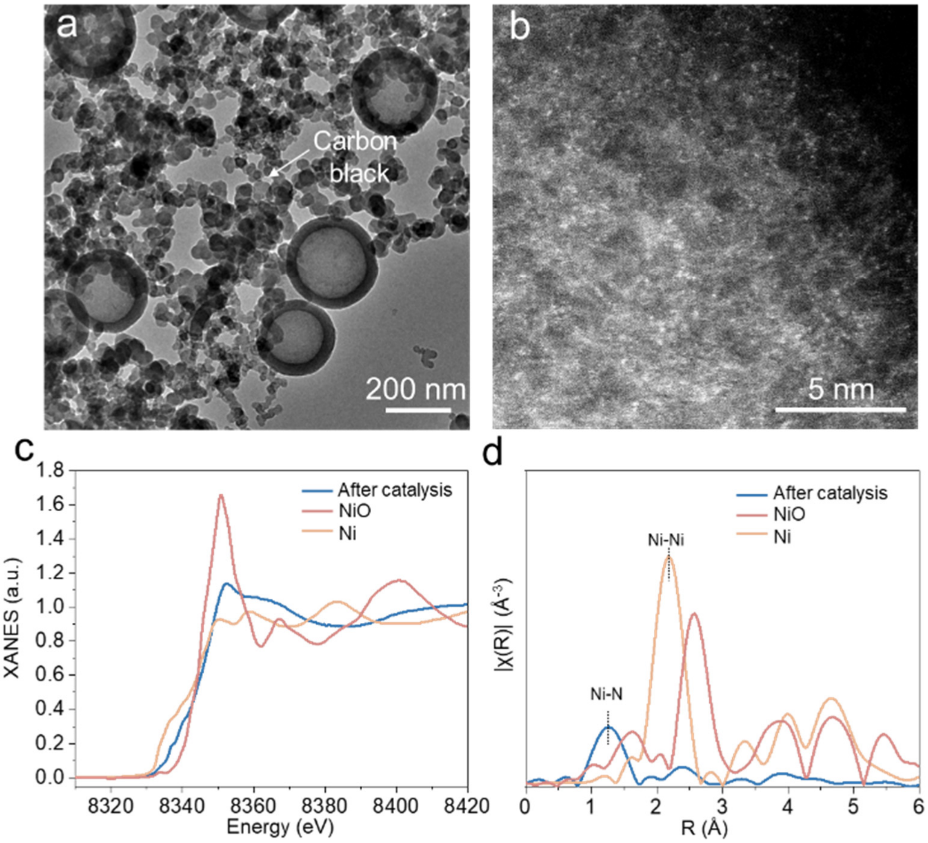

Supplementary Fig. 50 | Post stability test characterization of Ni-SAC-250. (a) TEM image, (b) AC-HAADF-STEM image, (c) Ni K-edge XANES spectra and (d) Ni K-edge Fourier-transformed (FT) k^2-weighted $\chi(k)$ functions.

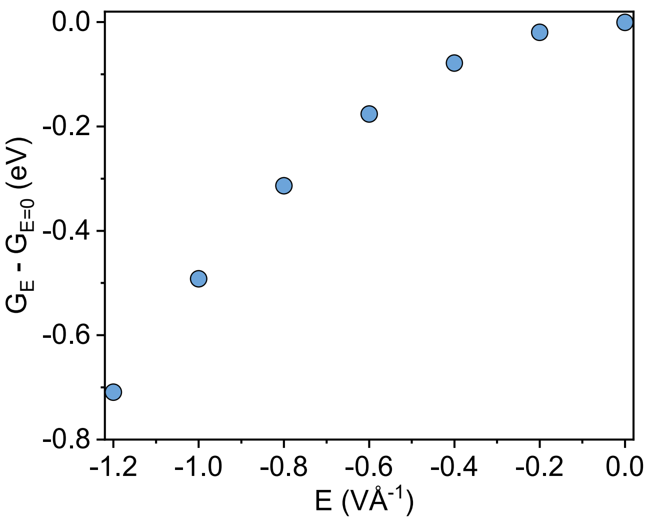

Supplementary Fig. 51 | The normalized total energy of the Ni-N_4 active site as a function of interfacial electric field strength.

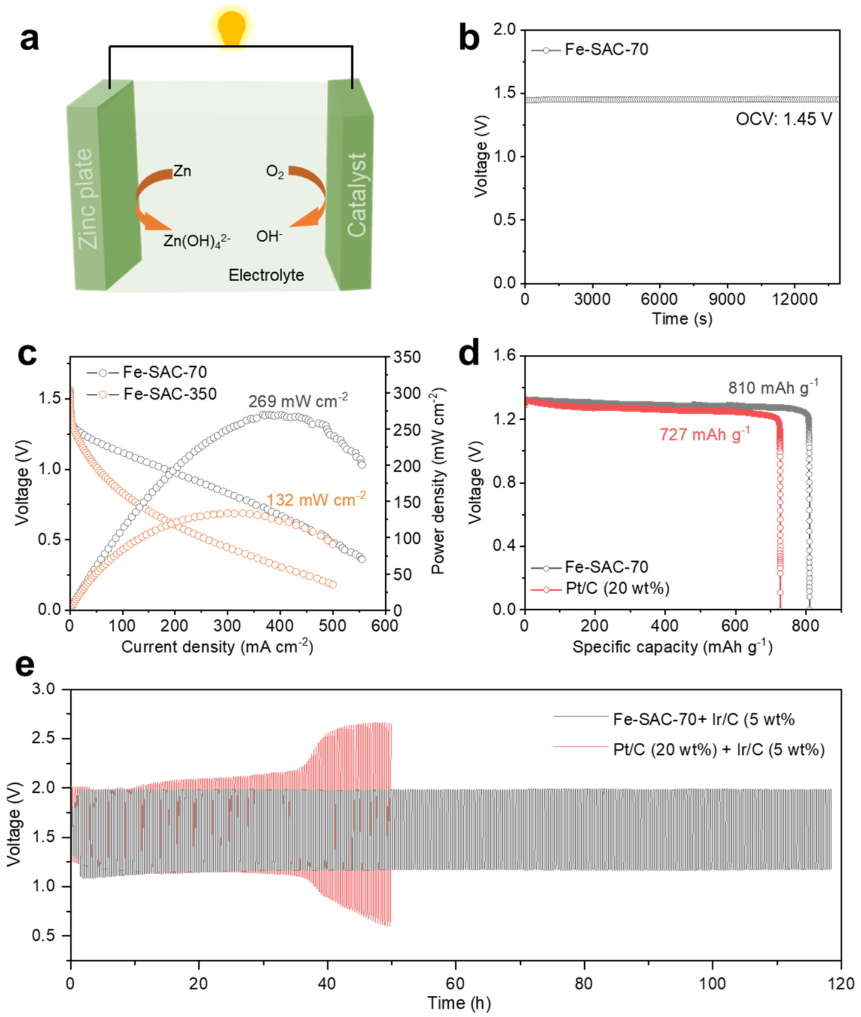

Supplementary Fig. 52 | (a) Schematic of the aqueous Zn-air battery. (b) OCV plot of the Zn-air battery with Fe-SAC-70 at 25°C. (c) Polarization and power density curves of the Zn-air battery with Fe-SAC-70 and Fe-SAC-350 as the cathodic catalysts. (d) Discharging capacity plots at a constant current density of 10 mA cm^{-2} for the Zn-air battery with Fe-SAC-70 and Pt/C (20 wt%) as the cathodic catalysts. (e) Charge-discharge profiles of two Zn–air batteries at a current density of 5 mA cm^{-2} with each cycle set to 10 min.

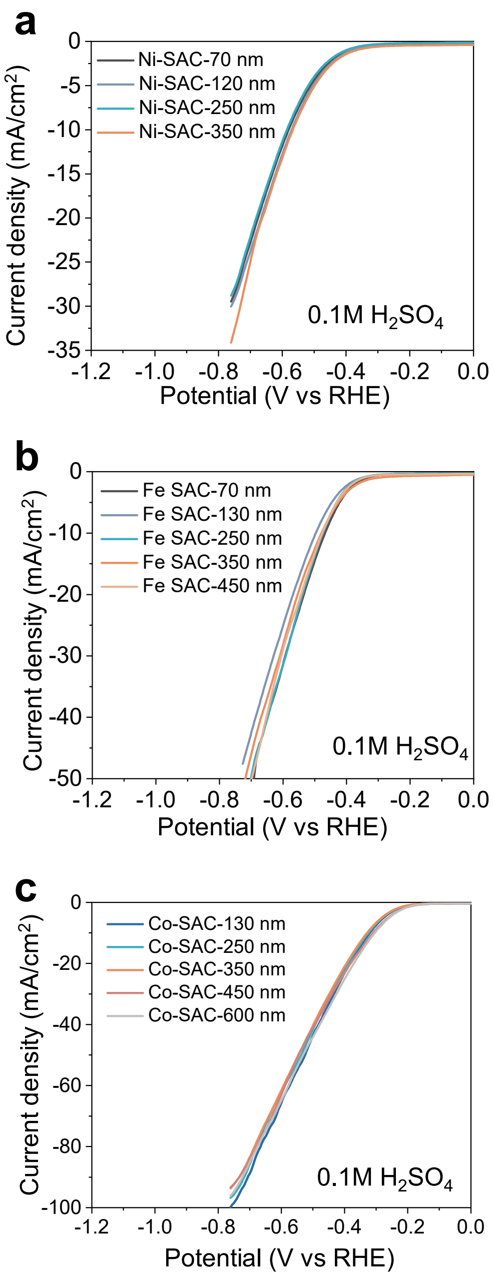

Supplementary Fig. 53 | HER LSV curves of (a) Ni-SACs, (b) Fe-SACs and (c) Co-SACs with different sphere diameters in 0.1 M H_2SO_4 electrolyte.

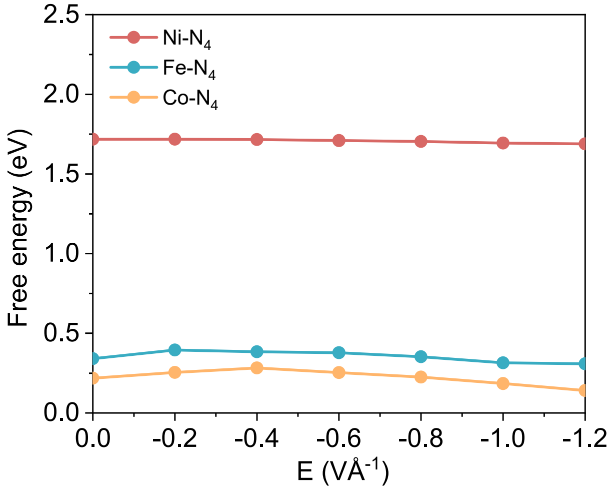

Supplementary Fig. 54 | *H adsorption energy as a function of electric field strength on Ni-N_4, Fe-N_4, and Co-N_4.

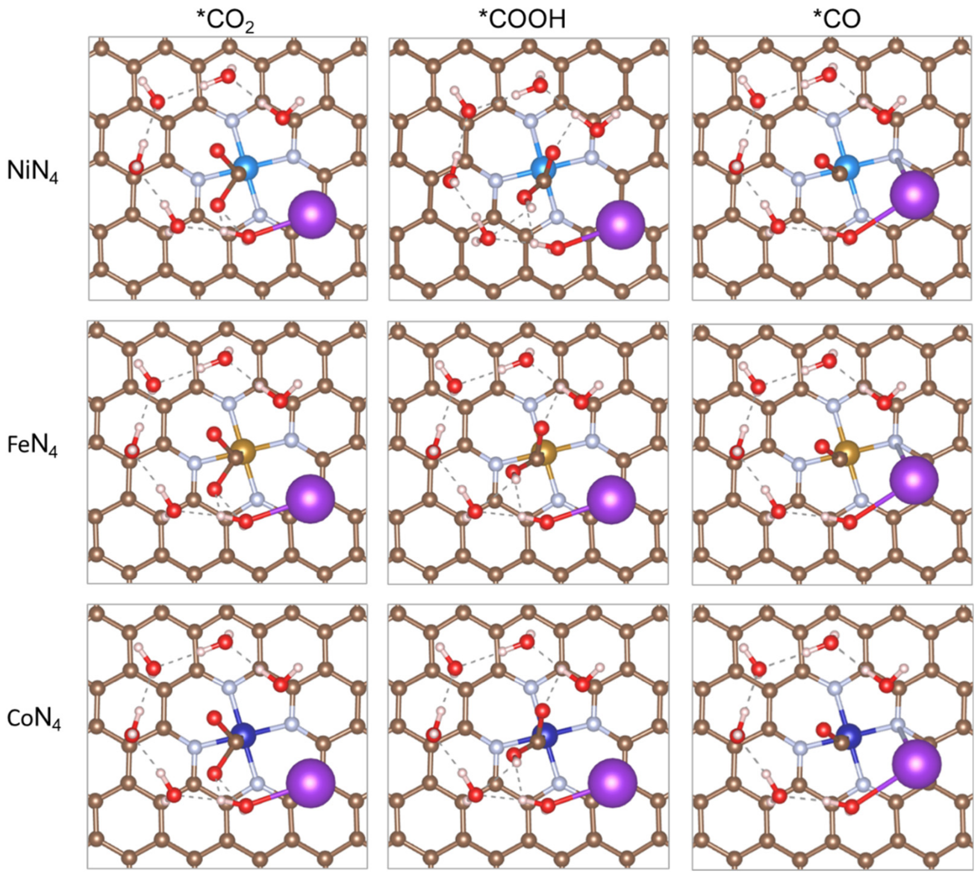

Supplementary Fig. 55 | Model structures of NiN_4, FeN_4 and CoN_4 with K^+ and H_2O absorbed with different CO_2R intermediates. The brown, gray, blue, yellow, dark blue, red, purple, and light pink spheres represent carbon, nitrogen, nickel, iron, cobalt, oxygen, potassium, and hydrogen atoms, respectively.

Supplementary Table 1 | Synthetic conditions for the hollow polymer nanospheres with different sphere diameters.

2,4-dihydroxy-benzoic acid (mmol)	Hexamethyl-enetetramine (mmol)	Sphere diameter (nm)
1.2	1.4	100±20
1.2	0.75	170±30
1.2	0.25	300±50
1.4	0.33	400±90
1.56	0.33	500±100
1.92	0.33	650±100

Supplementary Table 2 | ICP results of the different SACs studied in this work.

Catalysts	Metal loading Ni (wt.%)	Metal loading Co (wt.%)	Metal loading Fe(wt.%)
Ni-SAC-70 nm	0.82	--	--
Ni-SAC-130 nm	0.80	--	--
Ni-SAC-250 nm	0.81	--	--
Ni-SAC-350 nm	0.82	--	--
Co-SAC-130 nm	--	0.71	--
Co-SAC-350 nm	--	0.73	--
Co-SAC-450 nm	--	0.72	--
Co-SAC-600 nm	--	0.78	--
Fe-SAC-70 nm	--	--	0.60
Fe-SAC-250 nm	--	--	0.68
Fe-SAC-350 nm	--	--	0.65
Fe-SAC-450 nm	--	--	0.73

Supplementary Table 3 | Structural parameters of different Ni-SACs extracted from EXAFS fitting.

Samples	Path	CN	R (Å)	σ^2 (×10^{-3} Å^2)	ΔE_0 (eV)	R factor
Ni foil	Ni-Ni	12	2.48	5.9	-5.04	0.002
Ni-SAC-70 nm	Ni-N	4.2	1.81	8.2	-5.86	0.009
Ni-SAC-130 nm	Ni-N	4.1	1.82	5.6	-4.50	0.005
Ni-SAC-250 nm	Ni-N	4.0	1.81	6.5	-4.41	0.009
Ni-SAC-350 nm	Ni-N	4.1	1.81	7.1	-5.10	0.010

CN is the coordination number; R is interatomic distance (the bond length between central atoms and surrounding coordination atoms); σ^2 is Debye-Waller factor (a measure of thermal and static disorder in absorber-scatter distances); ΔE_0 is edge-energy shift (the difference between the zero kinetic energy value of the sample and that of the theoretical model). R factor is used to value the goodness of the fitting. Error bounds (accuracies) that characterize the structural parameters obtained by EXAFS spectroscopy were estimated as CN ± 20%; R± 1%; σ 2 ± 20%; ΔE_0 ± 20%. S_0^2 was set to 0.77, according to the experimental EXAFS fit of Ni foil reference by fixing CN as the known crystallographic value.

Supplementary Table 4 | Structural parameters of different Fe-SACs extracted from EXAFS fitting.

Samples	Path	CN	R (Å)	σ^2 ($\times10^{-3}$ Å^2)	ΔE_0 (eV)	R factor
Fe foil	Fe-Fe	8	2.46	3.4	2.7	0.007
	Fe-Fe	6	2.85	3.8		
Fe-SAC-70 nm	Fe-N	4.1	1.95	8.7	9.1	0.015
Fe-SAC-250 nm	Fe-N	4.3	1.94	8.1	5.6	0.016
Fe-SAC-350 nm	Fe-N	4.1	1.94	8.6	5.8	0.014
Fe-SAC-450 nm	Fe-N	4.1	1.94	7.1	3.8	0.015

CN is the coordination number; R is interatomic distance (the bond length between central atoms and surrounding coordination atoms); σ^2 is Debye-Waller factor (a measure of thermal and static disorder in absorber-scatter distances); ΔE_0 is edge-energy shift (the difference between the zero kinetic energy value of the sample and that of the theoretical model). R factor is used to value the goodness of the fitting. Error bounds (accuracies) that characterize the structural parameters obtained by EXAFS spectroscopy were estimated as CN ± 20%; R± 1%; σ 2 ± 20%; ΔE_0 ± 20%. S_0^2 was set to 0.78, according to the experimental EXAFS fit of Fe foil reference by fixing CN as the known crystallographic value.

Supplementary Table 5 | Structural parameters of different Co-SACs extracted from the EXAFS fitting.

Samples	Path	CN	R (Å)	σ^2 ($\times 10^{-3}$ Å^2)	ΔE_0 (eV)	R factor
Co foil	Co-Co	12	2.48	5.5	7.1	0.005
Co-SAC-130 nm	Co-N	4.1	1.86	9.7	9.3	0.016
	Co-C	3.0	2.91	3.5		
Co-SAC-350 nm	Co-N	3.9	1.86	9.3	7.0	0.016
	Co-C	3.1	2.92	3.6		
Co-SAC-450 nm	Co-N	4.1	1.87	7.5	9.4	0.018
	Co-C	3.5	2.90	4.3		
Co-SAC-600 nm	Co-N	4.3	1.86	6.4	6.3	0.015
	Co-C	2.7	2.92	3.5		

CN is the coordination number; R is interatomic distance (the bond length between central atoms and surrounding coordination atoms); σ^2 is Debye-Waller factor (a measure of thermal and static disorder in absorber-scatter distances); ΔE_0 is edge-energy shift (the difference between the zero kinetic energy value of the sample and that of the theoretical model). R factor is used to value the goodness of the fitting. Error bounds (accuracies) that characterize the structural parameters obtained by EXAFS spectroscopy were estimated as CN ± 20%; R± 1%; σ 2 ± 20%; ΔE_0 ± 20%. S_0^2 was set to 0.70, according to the experimental EXAFS fit of Co foil reference by fixing CN as the known crystallographic value.

Supplementary Table 6 | Faradaic efficiency data for Ni-SAC-70 in acidic electrolyte. Gas phase products H_2 and CO are shown here.

Current density (mA cm^{-2})	CO (%)	Stdev (%)	H_2 (%)	Stdev (%)
50	95.0	2.23	0.8	1.5
100	98.1	0.13	1.8	0.7
200	96.0	0.97	1.2	0.2
300	80.6	2.11	19.7	2.5
400	73.8	3.10	24.1	2.1

Supplementary Table 7 | Faradaic efficiency data for Ni-SAC-130 in acidic electrolyte. Gas phase products H_2 and CO are shown here.

Current density (mA cm^{-2})	CO (%)	Stdev (%)	H_2 (%)	Stdev (%)
50	94.0	1.54	4.3	0.5
100	97.9	2.08	3.1	1.8
200	96.8	3.01	1.2	0.16
300	90.0	1.15	9.9	0.8
400	81.2	1.49	19.0	1.2

Supplementary Table 8 | Faradaic efficiency data for Ni-SAC-250 in acidic electrolyte. Gas phase products H_2 and CO are shown here.

Current density (mA cm^{-2})	CO (%)	Stdev (%)	H_2 (%)	Stdev (%)
50	90.0	1.66	14.0	0.3
100	97.9	1.27	1.8	0.6
200	99.6	0.12	1.2	0.8
300	100.2	0.09	0.96	0.5
400	100.0	1.02	0.89	0.1

Supplementary Table 9 | Faradaic efficiency data for Ni-SAC-350 in acidic electrolyte. Gas phase products H_2 and CO are shown here.

Current density (mA cm^{-2})	CO (%)	Stdev (%)	H_2 (%)	Stdev (%)
50	84.0	2.78	13.0	0.2
100	95.2	0.84	1.8	0.6
200	96.1	0.97	1.2	0.8
300	98.0	2.28	0.96	0.7
400	90.2	2.32	9.0	1.7

Supplementary Table 10 | Comparison of CO_2R performance in acidic electrolyte between our Ni, Co and Fe-SACs with catalyst systems reported in the literature.

Catalysts	Catholyte	pH	FE_{co} (%)	j_{co}(mA cm^{-2})	Refs
Ni-N-C	0.5 M K_2SO_4+ H_2SO_4	1	82	410	*Energy Environ. Sci.*, 2023,**16**, 1502-1510[7]
Au/Carbon	0.4 M K_2SO_4+ H_2SO_4	1.5	91	227	*Nat. Catal.* 2022, **5**, 268−276[8]
Ag powder	1 M C_2SO_4+ H_2SO_4	0.5	52.5	105	*ACS Energy Lett.* 2022, **7**, 4224−4231[9]
Ni-N-C/PTFE	1 M C_2SO_4+ H_2SO_4	2	100	250	*Adv. Mater.* 2022, **34**, e2201295[10]
Au nanoparticles	1 M Cs_2SO_4+ H_2SO_4	3	80	160	*Nat. Commun.* 2021, **12**, 4943[11]
Fe-N-C	0.1 M H_3PO_4-K_3PO_4	2	10	1.3	*ACS Energy Lett.* 2018, **3**, 812−817[12]
Co protoporphyrin	0.1 M $NaClO_4$+ $HClO_4$	1	0.6	0.17	*Nat Commun.* 2015, **6**, 8177[13]
Ni-SAC-250	0.1 M K_2SO_4+ H_2SO_4	1	＞99	400	**This work**
Fe-SAC-350	0.1 M K_2SO_4+ H_2SO_4	1	88.5	88.5	**This work**
Co-SAC-450	0.1 M K_2SO_4+ H_2SO_4	1	56	56	**This work**

Supplementary Table 11 | Faradaic efficiency data for Fe-SAC-130 in acidic electrolyte. Gas phase products H_2 and CO are shown here.

Current density (mA cm^{-2})	CO (%)	Stdev (%)	H_2 (%)	Stdev (%)
50	64.8	4.65	33.2	2.3
100	65.0	5.01	33.4	4.8
150	21.6	3.43	75.1	5.8
200	11.4	1.09	87.4	2.8
300	6.9	0.46	91.4	1.8
400	4.67	0.81	94.9	4.2

Supplementary Table 12 | Faradaic efficiency data for Fe-SAC-250 in acidic electrolyte. Gas phase products H_2 and CO are shown here.

Current density (mA cm^{-2})	CO (%)	Stdev (%)	H_2 (%)	Stdev (%)
50	63.2	5.4	39.1	5.1
100	83.1	4.1	14.4	9.8
150	44.1	3.8	53.0	6.1
200	28.6	2.8	68.4	3.0
300	16.7	1.7	80.5	1.8
400	8.0	0.75	90.1	5.4

Supplementary Table 13 | Faradaic efficiency data for Fe-SAC-350 in acidic electrolyte. Gas phase products H_2 and CO are shown here.

Current density (mA cm^{-2})	CO (%)	Stdev (%)	H_2 (%)	Stdev (%)
50	80.2	0.5	19.9	0.2
100	88.5	2.5	12.4	3.0
150	65.5	4.1	36.2	8.3
200	40.1	5.8	57.8	5.0
300	18.2	3.7	80.2	3.9
400	10.4	0.9	85.0	1.4

Supplementary Table 14 | Faradaic efficiency data for Fe-SAC-450 in acidic electrolyte. Gas phase products H_2 and CO are shown here.

Current density (mA cm^{-2})	CO (%)	Stdev (%)	H_2 (%)	Stdev (%)
50	79.2	3.6	11.9	1.4
100	72.9	5.8	27.2	2.5
150	42.8	4.9	54.3	1.2
200	27.3	3.7	70.0	7.6
300	14.2	2.6	79.1	2.1
400	8.1	1.3	89.5	2.2

Supplementary Table 15 | Faradaic efficiency data for Co-SAC-250 in acidic electrolyte. Gas phase products H_2 and CO are shown here.

Current density (mA cm^{-2})	CO (%)	Stdev (%)	H_2 (%)	Stdev (%)
50	37.7	3.9	59.2	3.6
100	41.3	2.8	57.2	2.5
150	31.7	2.4	65.6	3.9
200	31.8	1.1	66.0	3.6
300	24.5	0.5	72.3	2.4
400	15.0	0.4	81.6	3.2

Supplementary Table 16 | Faradaic efficiency data for Co-SAC-350 in acidic electrolyte. Gas phase products H_2 and CO are shown here.

Current density (mA cm^{-2})	CO (%)	Stdev (%)	H_2 (%)	Stdev (%)
50	41.7	2.3	54.5	1.5
100	48.3	0.8	52.1	1.2
150	39.3	3.4	60.2	1.5
200	37.3	2.1	59.1	0.6
300	27.1	2.2	68.4	3.4
400	19.8	2.1	78.6	2.9

Supplementary Table 17 | Faradaic efficiency data for Co-SAC-450 in acidic electrolyte. Gas phase products H_2 and CO are shown here.

Current density (mA cm^{-2})	CO (%)	Stdev (%)	H_2 (%)	Stdev (%)
50	51.8	2.3	47.3	1.7
100	56.0	0.8	44.1	0.6
150	54.6	3.4	47.1	1.4
200	48.9	2.1	51.7	2.0
300	37.9	2.2	59.7	0.8
400	21.3	2.1	75.9	1.5

Supplementary Table 18 | Faradaic efficiency data for Co-SAC-600 in acidic electrolyte. Gas phase products H_2 and CO are shown here.

**Current density (mA cm^{-2})**	**CO (%)**	**Stdev (%)**	**H_2 (%)**	**Stdev (%)**
50	18.8	1.0	78.3	3.6
100	27.3	0.9	69.4	3.5
150	16.2	1.5	76.7	1.4
200	10.6	0.9	86.2	2.3
300	6.6	2.6	87.9	1.1
400	4.6	1.25	91.9	2.5

Supplementary Table 19 | Comparison of ORR performance in 0.1 M KOH between Fe-SAC-70 and reported Fe-based SACs in the literature.

Catalysts	E_{onset} (V)	$E_{1/2}$(V)	Refs
Fe-SAC-70	1.02	0.91	**This work**
MnFeCoNiCu-HESA	0.92	0.84	*Nat. Sustain.,* 2023, *doi.org/10.1038/s41893-023-01101-z*[14]
FePc/GaS	0.97	0.895	*Angew. Chem. Int. Ed.* 2023, **62**, e202212335[15]
MQFe-10:20:5	0.96	0.86	*Angew. Chem. Int. Ed.* 2022, **134**, e202117617[16]
FeN_4-Te_n	0.92	0.87	*Adv. Mater.* 2022, **34**, 2202714[17]
FeSA-N-C	0.98	0.90	*Nat. Commun.* 2020, **11**, 2831[18]
Fe-N/P-C-700	0.94	0.87	*J. Am. Chem. Soc.* 2020, **142**, 2404[19]
Fe_1-HNC-500-850	NA	0.84	*Adv. Mater.* 2020, **32**, 1906905[20]
Fe-NC SAC	0.98	0.90	*Nat. Commun.* 2019, **10**, 1278[21]
Fe-N-C HNSs	1.06	0.87	*Adv. Mater.* 2019, **31**, 1806312[22]
Fe-SAs/NSC	1.00	0.87	*J. Am. Chem. Soc.* 2019, **141**, 20118[23]
Fe SAC/N-C	1.01	0.89	*Adv. Mater.* 2019, **31**, 1808193[24]
Fe_{SA}–N–C	N.A.	0.89	*Angew. Chem. Int. Ed.* 2018, **57**, 8525[25]
SA-Fe/NG	1.0	0.88	*Proc. Natl. Acad. Sci. U.S.A* 2018, **115**, 6626[26]

Supplementary Table 20 | Comparison of Zn-air battery performance of Fe-SAC-70 with previously reported state of art catalysts.

Catalysts	OCV (V)	Specfic capacity (mAh/gZn)	Peak Power density (mW/cm^2)	Refs
Fe-SAC-70	1.45	810	269	**This work**
MnFeCoNiCu-HESA	1.48	779	207	*Nat. Sustain.,* 2023, *doi.org/10.1038/s41893-023-01101-z*[14]
Fe-Se/NC	NA	764	135	*Angew. Chem. Int. Ed.* 2023, **61**, e202115219[27]
Fe,Mn/NC	1.40	902	160.8	*Nat. Commun.* 2021, **12**, 1734[28]
SA-Fe-NCPC	1.516	795.3	266.4	*Adv. Mater.* 2020, **32**, 1907399[29]
Fe/Ni-N_x/OC	1.525	712	148	*Adv. Mater.* 2020, **32**, 2004670[30]
Fe-N/P-C-700	1.42	723.6	133.2	*J. Am. Chem. Soc.* 2020, **142**, 2404[19]
PdMo bimetallene	1.48	798	154.2	*Nature* 2019*, **574**,* 81[31]
Fe-SAs/NPS-HC	1.45	N.A.	195	*Nat. Commun.,* 2018, **9**, 5422[32]

Supplementary Note 1

The finite element numerical method simulations of the electric field are implemented within COMSOL Multiphysics. It is a stationary, isothermal and 1D symmetric model, which consists of a hollow carbon nanosphere, meshed by a conventional free triangular operation. The electrolyte was set as 0.1 M K_2SO4 and 0.05 M H_2SO_4. The automatic highly nonlinear method was used in the stationary solver. Electrostatics, electric current and transport of diluted species modules were coupled to solve the charge density, ion concentration and electric field.

The electric current module can compute the electric field distribution under the applied potential, which was set as a boundary condition at a value of -0.8V in this model. The electric field was calculated by:

$$E = -\nabla V$$

And the free charge density was calculated by:

$$\nabla \cdot D = \rho_V$$

where E is the electric field, V is the potential, ρ_V is the space charge density, and the electric displacement D follows:

$$D = \varepsilon_0 \varepsilon_r E$$

where ε_0 is the dielectric constant for vacuum, and ε_r is the dielectric constant for a material.

In the Gouy-Chapman-Stern model of the electric double layer, the thickness of the Helmholtz layer (Stern layer) was identical to the radius of a hydrated ion. Based on the Poisson-Nernst-Planck equations, in the Helmholtz layer,

$$\nabla^2 V = 0$$

And in the diffuse layer,

$$\nabla^2 V = \rho_V = F(c_+ - c_-)$$

The mass flux of species in the electrolyte n_i was calculated by:

$$n_i = -D_i \nabla c_i - z_i u_{m,i} F c_i \nabla V$$

where D_i is the diffusivity of the chemical species, c_i is the concentration of species, z_i is the valence of ionic species, $u_{m,i}$ is the mobility in medium m and F is the Faraday's constant.

In the model, triangular meshes were applied with a maximum mesh size of 200nm, a minimum size of 0.1 nm and a maximum growth rate of 1.3. Hence, for a sphere with a radius of 50 nm, the mesh number is 3318.

The mesh sensitivity analysis has been carried out by performing the same simulation

with different mesh settings. With a smaller maximum mesh size and maximum element growth rate, the number of elements increased but did not influence the simulation solution, indicating that the mesh created was good enough to ensure convergence of numerical results.

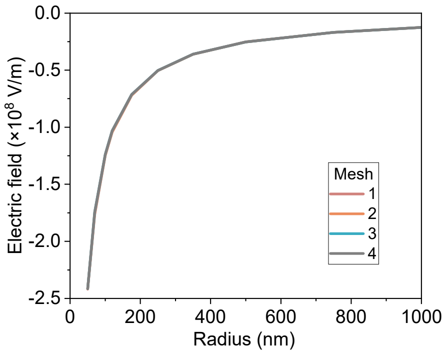

Supplementary Fig. 50 | The electric field distribution vs sphere radius under different mesh sizes.

Supplementary Table 21. Parameters of each different mesh.

Mesh	Maximum mesh size	Minimum mesh size	Maximum growth rate
1	200	0.08	1.1
2	200	0.1	1.3
3	400	1	1.3
4	800	10	1.3

For the purposes of this work, a parametric sweep was used with different radius of the nanosphere (50, 70, 100, 120, 175, 250, 350, 500, 750 and 1000 nm). The electric field distribution over the surface of the carbon nanosphere could be solved correspondingly.

Physical constants

c_i Concentration, mol/m^3
D Electric displacement, C/m^2
D_i Diffusivity, m^2/s
E Electric field, V/m
F Faraday's constant, C/mol

n_i Mass flux, g/(m^2·s)

$u_{m,i}$ Mobility in medium m, s/(mol·kg)

V Potential, V
z_i Valence of ionic species

ε_0 Dielectric constant for vacuum, C/(V·m)

ε_r Dielectric constant for a material, C/(V·m)

ρ_V Space charge density, C/m^3

Supplementary Note 2

TOF calculations (CO_2R)

For CO_2R, the TOFs are calculated based on the following equation:

$$\mathrm{TOF}(h^{-1}) = \frac{i_{product}/2F}{m_{cat} * w/M_{metal}} * 3600$$

Where the *i* is the partial current of a specific product of CO or H_2, A; *F* is the Faradaic constant, 96485 C mol^{-1}; m_{cat} is the mass of the catalyst on the electrode, g; *w* is the metal loading in the catalyst based on the ICP-OES; the M_{metal} is the atomic mass, Ni (58.69 g mol^{-1}), Fe (55.85 g mol^{-1}), Co (58.93 g mol^{-1}).

TOF calculations (OER and HER)

For OER and HER, the TOFs calculated based on the following equation:

$$\mathrm{TOF}(h^{-1}) = \frac{i}{n * F * m} * 3600$$

Where the *i* is the current measured on the LSV curve at a given potential, A; *F* is the Faradaic constant, 96485 C mol^{-1}; *m* is the mole amount of metal single atoms on the electrode, mol; n is the electron transfer number, for OER is 4 and HER is 2 based on the equation of $4OH^- - 4e^- \rightarrow O_2 + 2H_2O$ and $2H^+ + 2e^- \rightarrow H_2$, respectively.

Supplementary Note 3

CO_2R studies with Ni-N_4, Fe-N_4 and Co-N_4

We use density functional theory (DFT) and an explicit model of the electrochemical interface to investigate the energetics of the $CO_2 \rightarrow CO$ pathway on Ni-N_4, Fe-N_4 and Co-N_4. Under electrochemical conditions, we show that the adsorbed intermediates are significantly affected by local electric fields at the electrode/electrolyte interface.

We study the following reaction pathway[33]:

$$CO_2(g) + * \rightarrow *CO_2$$
$$*CO_2 + H_2O + e^- \rightarrow *COOH + OH^-$$
$$*COOH + H_2O + e^- \rightarrow *CO + H_2O + OH^-$$
$$*CO \rightarrow CO(g) + *$$

where a lone asterisk (*) denotes a surface site and an * symbol before a molecule denotes an adsorbed species.

ORR studies with Fe-N_4

The ORR reaction pathway on Fe-N_4 was calculated. The computational hydrogen electrode was utilized to obtain free energies for each state as in ref[34]. The four-electron ORR pathway could be summarized by the following elementary steps[35]:

$$O_2(g) + 2H_2O(l) + 4e^- + * \rightarrow OOH* + OH^- + H_2O(l) +3e^-$$
$$OOH* + OH^- + H_2O(l) + 3e^- \rightarrow O* + 2OH^- + H_2O(l) +2e^-$$
$$O* + 2OH^- +H_2O(l) + 2e^- \rightarrow OH* + 3OH^- + e^-$$
$$OH* + 3OH^- + e^- \rightarrow 4OH^- + *$$

The free energies of reactants and each intermediate state at an applied electrode potential U were calculated as follows: $G(U) = \Delta E + \Delta ZPE - T\Delta S - neU$, where n is the electron number of such state and ΔE represents the change in enthalpy, which is considered from the DFT total energy value, ΔZPE represents the change in zero-point energy and ΔS represents the change in entropy. Since it is difficult to obtain the exact free energy of OOH, O, and OH radicals in the electrolyte solution, the adsorption free energy ΔG_{OOH*}, ΔG_{O*}, and ΔG_{OH*} are used in the calculations.

At equilibrium potential U^0, the free energy change of four steps could be obtained as follows:

$$\Delta G_A(U^0) = G_{OOH*} + G_{H2O(l)} -G* - 3G_{OH-} -3eU^0$$
$$\Delta G_B(U^0) = G_{O*} - G_{OOH*} + G_{OH-} - eU^0$$
$$\Delta G_C(U^0) = G_{OH*} - G_{H2O(l)} - G_{O*} + G_{OH-} - eU^0$$

$$\Delta G_D(U^0) = -G_{OH*} + G_{O*} + G_{OH-} + eU^0$$

The equilibrium potential U^0 for ORR was determined to be 1.229 V vs SHE where the reactant and product are at the same energy level.

Supplementary References

1 Wan, X. *et al.* Iron atom–cluster interactions increase activity and improve durability in Fe–N–C fuel cells. *Nat. Commun.* **13**, 2963 (2022).

2 Wang, X. *et al.* Insight into dynamic and steady-state active sites for nitrogen activation to ammonia by cobalt-based catalyst. *Nat. Commun.* **11**, 653 (2020).

3 Wang, Q. *et al.* Attenuating metal-substrate conjugation in atomically dispersed nickel catalysts for electroreduction of CO_2 to CO. *Nat. Commun.* **13**, 6082 (2022).

4 Ren, X. *et al.* In-situ spectroscopic probe of the intrinsic structure feature of single-atom center in electrochemical CO/CO_2 reduction to methanol. *Nat. Commun.* **14**, 3401 (2023).

5 Zhang, Z., Zhu, J., Chen, S., Sun, W. & Wang, D. Liquid fluxional Ga single atom catalysts for efficient electrochemical CO_2 reduction. *Angew. Chem. Int. Ed.* **62**, e202215136 (2023).

6 Zhang, L. *et al.* Atomically dispersed Ni-Cu catalysts for pH-universal CO_2 electroreduction. *Adv. Mater.*, 2209590 (2023).

7 Li, H. *et al.* Tailoring acidic microenvironments for carbon-efficient CO_2 electrolysis over a Ni–N–C catalyst in a membrane electrode assembly electrolyzer. *Energy Environ. Sci.* **16**, 1502-1510 (2023).

8 Gu, J. *et al.* Modulating electric field distribution by alkali cations for CO_2 electroreduction in strongly acidic medium. *Nat. Catal.* **5**, 268-276 (2022).

9 Pan, B. *et al.* Close to 90% single-pass conversion efficiency for CO_2 electroreduction in an acid-fed membrane electrode assembly. *ACS Energy Lett.* **7**, 4224-4231 (2022).

10 Sheng, X., Ge, W., Jiang, H. & Li, C. Engineering the Ni-N-C catalyst microenvironment enabling CO_2 electroreduction with nearly 100% CO selectivity in acid. *Adv. Mater.* **34**, 2201295 (2022).

11 Monteiro, M. C., Philips, M. F., Schouten, K. J. P. & Koper, M. T. Efficiency and selectivity of CO_2 reduction to CO on gold gas diffusion electrodes in acidic media. *Nat. Commun.* **12**, 4943 (2021).

12 Varela, A. S. *et al.* pH effects on the selectivity of the electrocatalytic CO_2 reduction on graphene-embedded Fe–N–C motifs: bridging concepts between molecular homogeneous and solid-state heterogeneous catalysis. *ACS Energy Lett.* **3**, 812-817 (2018).

13 Shen, J. *et al.* Electrocatalytic reduction of carbon dioxide to carbon monoxide and methane at an immobilized cobalt protoporphyrin. *Nat. Commun.* **6**, 8177 (2015).

14 Lei, X. *et al.* High-entropy single-atom activated carbon catalysts for sustainable oxygen electrocatalysis. *Nat. Sustain.*, 1-11 (2023).

15 Zhuang, Z. *et al.* Continuous modulation of electrocatalytic oxygen reduction activities of single-atom catalysts through p-n junction rectification. *Angew. Chem. Int. Ed.* **62**, e202212335 (2023).

16 Li, M., Lv, Q., Si, W., Hou, Z. & Huang, C. Sp-hybridized nitrogen as new anchoring sites of iron single atoms to boost the oxygen reduction reaction. *Angew. Chem. Int. Ed.* **61**, e202208238 (2022).

17 Ji, B. *et al.* Metalloid-cluster ligands enabling stable and active FeN_4-Te_n motifs for the oxygen reduction reaction. *Adv. Mater.* **34**, 2202714 (2022).

18 Jiao, L. *et al.* Nanocasting SiO_2 into metal–organic frameworks imparts dual protection to high-loading Fe single-atom electrocatalysts. *Nat. Commun.* **11**, 2831 (2020).

19 Yuan, K. *et al.* Boosting oxygen reduction of single iron active sites via geometric and electronic engineering: nitrogen and phosphorus dual coordination. *J. Am. Chem. Soc.* **142**, 2404-2412 (2020).

20 Zhang, X. *et al.* A general method for transition metal single atoms anchored on honeycomb-like nitrogen-doped carbon nanosheets. *Adv. Mater.* **32**, 1906905 (2020).

21 Zhao, L. *et al.* Cascade anchoring strategy for general mass production of high-loading single-atomic metal-nitrogen catalysts. *Nat. Commun.* **10**, 1278 (2019).

22 Chen, Y. *et al.* Atomic Fe dispersed on N-doped carbon hollow nanospheres for high-efficiency electrocatalytic oxygen reduction. *Adv. Mater.* **31**, 1806312 (2019).

23 Zhang, J. *et al.* Tuning the coordination environment in single-atom catalysts to achieve highly efficient oxygen reduction reactions. *J. Am. Chem. Soc.* **141**, 20118-20126 (2019).

24 Lin, Y. *et al.* Fabricating single-atom catalysts from chelating metal in open frameworks. *Adv. Mater.* **31**, 1808193 (2019).

25 Jiao, L. *et al.* From metal–organic frameworks to single-atom Fe implanted N-doped porous carbons: efficient oxygen reduction in both alkaline and acidic media. *Angew. Chem. Int. Ed.* **130**, 8661-8665 (2018).

26 Yang, L. *et al.* Unveiling the high-activity origin of single-atom iron catalysts for oxygen reduction reaction. *Proc. Natl. Acad. Sci. U.S.A* **115**, 6626-6631 (2018).

27 Wang, Y. *et al.* Synergistic Fe−Se atom pairs as bifunctional oxygen electrocatalysts boost low-temperature rechargeable Zn-Air battery. *Angew. Chem. Int. Ed.* **62**, e202219191 (2023).

28 Yang, G. *et al.* Regulating Fe-spin state by atomically dispersed Mn-N in Fe-NC catalysts with high oxygen reduction activity. *Nat. Commun.* **12**, 1734 (2021).

29 Chen, G. *et al.* Zinc-mediated template synthesis of Fe-N-C electrocatalysts with densely accessible Fe-Nx active sites for efficient oxygen reduction. *Adv. Mater.* **32**, 1907399 (2020).

30 Zhu, Z. *et al.* Coexisting single-atomic Fe and Ni sites on hierarchically ordered porous carbon as a highly efficient ORR electrocatalyst. *Adv. Mater.* **32**, 2004670 (2020).

31 Luo, M. *et al.* PdMo bimetallene for oxygen reduction catalysis. *Nature* **574**, 81-85 (2019).

32 Chen, Y. *et al.* Enhanced oxygen reduction with single-atomic-site iron catalysts for a zinc-air battery and hydrogen-air fuel cell. *Nat. Commun.* **9**, 5422 (2018).

33 Chen, L. D., Urushihara, M., Chan, K. & Nørskov, J. K. Electric field effects in electrochemical CO_2 reduction. *ACS Catal.* **6**, 7133-7139 (2016).

34 Nørskov, J. K. *et al.* Origin of the overpotential for oxygen reduction at a fuel-cell cathode. *J. Phys. Chem. B* **108**, 17886-17892 (2004).

35 Jiao, Y., Zheng, Y., Jaroniec, M. & Qiao, S. Z. Origin of the electrocatalytic oxygen reduction activity of graphene-based catalysts: a roadmap to achieve the best performance. *J. Am. Chem. Soc.* **136**, 4394-4403 (2014).
